# Physical exercise for prevention of dementia study: a 48-month randomized controlled trial

**DOI:** 10.3389/fragi.2026.1847451

**Published:** 2026-08-10

**Authors:** Enzo Iuliano, Giovanni Fiorilli, Antonella Angiolillo, Graziamaria Corbi, Giulia Di Martino, Santina Ciccotelli, Carlo della Valle, Giuseppe Calcagno, Alessandra di Cagno, Alfonso Di Costanzo

**Affiliations:** 1 Centre for Research and Training in Medicine of Ageing, Department of Medicine and Health Sciences “V. Tiberio”, University of Molise, Campobasso, Italy; 2 Department of Theoretical and Applied Sciences, eCampus University, Novedrate, Italy; 3 Department of Medicine and Health Sciences “V. Tiberio”, University of Molise, Campobasso, Italy; 4 Department of Translational Medical Sciences, University of Naples “Federico II”, Naples, Italy; 5 Department of Human Sciences, Guglielmo Marconi University, Rome, Italy; 6 Department of Neurosciences, Biomedicine and Movement, University of Verona, Verona, Italy; 7 Department of Movement, Human and Health Sciences, University of Rome “Foro Italico”, Rome, Italy

**Keywords:** aging, clinical trial, cognitive decline, dementia, global health, neurology, physical activity, prevention strategies

## Abstract

**Background:**

Although multimodal exercise interventions are recognized for their potential to attenuate age-related cognitive decline, the current literature lacks longitudinal depth. Specifically, robust randomized evidence addressing the long-term efficacy of structured training protocols remains limited. This single-blind RCT involved 882 community-dwelling older adults categorized as healthy, having subjective memory complaints (SMCs), or having mild cognitive impairment (MCI).

**Methods:**

Participants were randomized to an experimental group (EG), performing 48 months of supervised moderate-to-high-intensity multimodal training (aerobic, resistance, and stretching), or to a control group (CG), maintaining usual habits. The primary outcome was the 48-month change in Mini-Mental State Examination (MMSE) scores. Secondary outcomes were scores on eight neuropsychological tests assessed at 12, 24, 36, and 48 months. A linear mixed-effects model analysis with repeated measures (LMM) was used to evaluate significant differences between the CG and EG.

**Results:**

In the final analysis of 674 participants, healthy participants (between-group effect: *p* = 0.008; η^2^_p_ = 0.007; time*group interaction: *p* < 0.001; η^2^_p_ = 0.025) in the EG showed significantly higher MMSE scores than those in the CG. SMC participants in the EG also showed higher MMSE scores than those in the CG (between-group effect: *p* = 0.052; η^2^_p_ = 0.004; time*group interaction: *p* < 0.001; η^2^p = 0.031), although the between-group difference only approached statistical significance. Similarly, healthy and SMC participants in the EG demonstrated significantly better performance in the secondary outcomes of memory, attention, and reasoning than those in the CG. The sample size of the MCI group was insufficient to allow for conclusive statistical analyses.

**Conclusion:**

A 48-month multicomponent exercise program significantly improved cognitive performance in healthy and SMC older adults. Structured physical activity may effectively slow cognitive decline.

**Clinical Trial Registration:**

https://clinicaltrials.gov/study/NCT02236416. Identifier number: NCT02236416.

## Introduction

Over the next 30 years, the number of people living with dementia worldwide is expected to be almost triple, with significant consequences for individuals, their families, public health, social care, and economy (Nichols et al., 2022). Unfortunately, medical treatments for any neurodegenerative dementia, including Alzheimer’s disease (AD), are shown to be not so effective in curing or delaying cognitive decline (Cummings et al., 2023; Kumaran et al., 2023), while preventive strategies might be a plausible strategy to prevent or delay the transition to dementia (Rao et al., 2023; Livingston et al., 2020). Fourteen modifiable lifestyle risk factors, including physical inactivity, account for 45% of all types of dementia worldwide that could potentially be prevented or delayed (Livingston et al., 2020). Preventive strategies are probably much more effective when implemented during the clinical stages preceding dementia, i.e., of subjective memory complaints (SMCs) or mild cognitive impairment (MCI) (Flier et al., 2023).

Regular physical activity (PA) is a very promising intervention as it could delay cognitive decline and dementia with minimal side effects and also have a positive influence on several chronic diseases associated with cognitive impairment, such as cardiovascular disease, hypertension, diabetes mellitus, and obesity (Livingston et al., 2020; Morton et al., 2022). Several mechanisms explain the protective role of physical exercise against dementia. Regular physical activity is associated with improved cerebral blood flow and vascular health, attenuation of hypoxia-related neuroinflammation, and the promotion of neuroplasticity and neurogenesis, particularly in the hippocampus, through the upregulation of neurotrophic factors. Moreover, exercise is associated with the preservation of brain structure and function, partly via myokine-mediated neuroprotection, and may contribute to the maintenance of cognitive reserve (Kaufman et al., 2024).

The efficacy of PA in preserving cognitive function appears to be moderated by an individual’s baseline cognitive status. Previous meta-analyses and umbrella reviews investigating cognitively healthy older adults have generally reported modest but significant neurocognitive improvements following structured exercise interventions (Demurtas et al., 2020; López et al., 2023; Yan et al., 2023). In contrast, evidence regarding individuals experiencing subjective memory complaints (SMCs) is still emerging; recent network meta-analyses suggest that structured exercise is effective in enhancing or maintaining memory during this specific prodromal stage (Chen et al., 2024). Similarly, among populations exhibiting objective cognitive decline, specifically those with mild cognitive impairment (MCI), the literature remains highly conflicting. Recent state-of-the-art reviews (Rookes et al., 2026) indicate potential neuroprotective benefits in MCI cohorts; yet, other systematic analyses conclude that the available data remain insufficient to establish robust clinical guidelines for these high-risk populations (Steichele et al., 2022; Liu et al., 2022; Ciria et al., 2023). However, nearly all studies have concluded that additional investigations are required.

Critical unanswered questions revolve around the parameters of exercise prescription, including frequency, intensity, duration, type, amount, and progression rate, which are essential for successful PA interventions (Demurtas et al., 2020; López et al., 2023). Therefore, randomized controlled trials (RCTs) with high methodological quality, including better structured PA programs, large sample sizes, and long-term follow-ups, are highly needed.

The present study was an RCT, named EPD (Physical Exercise for Prevention of Dementia), which was designed and conducted to determine whether a 48-month program of supervised and standardized multicomponent exercise could modify cognitive decline in older adults who were cognitively healthy or at risk of dementia.

## Methods

The EPD study protocol was previously published in detail (Iuliano et al., 2019). Below is the summary of the study methods.

### Trial design

**FIGURE 1:**
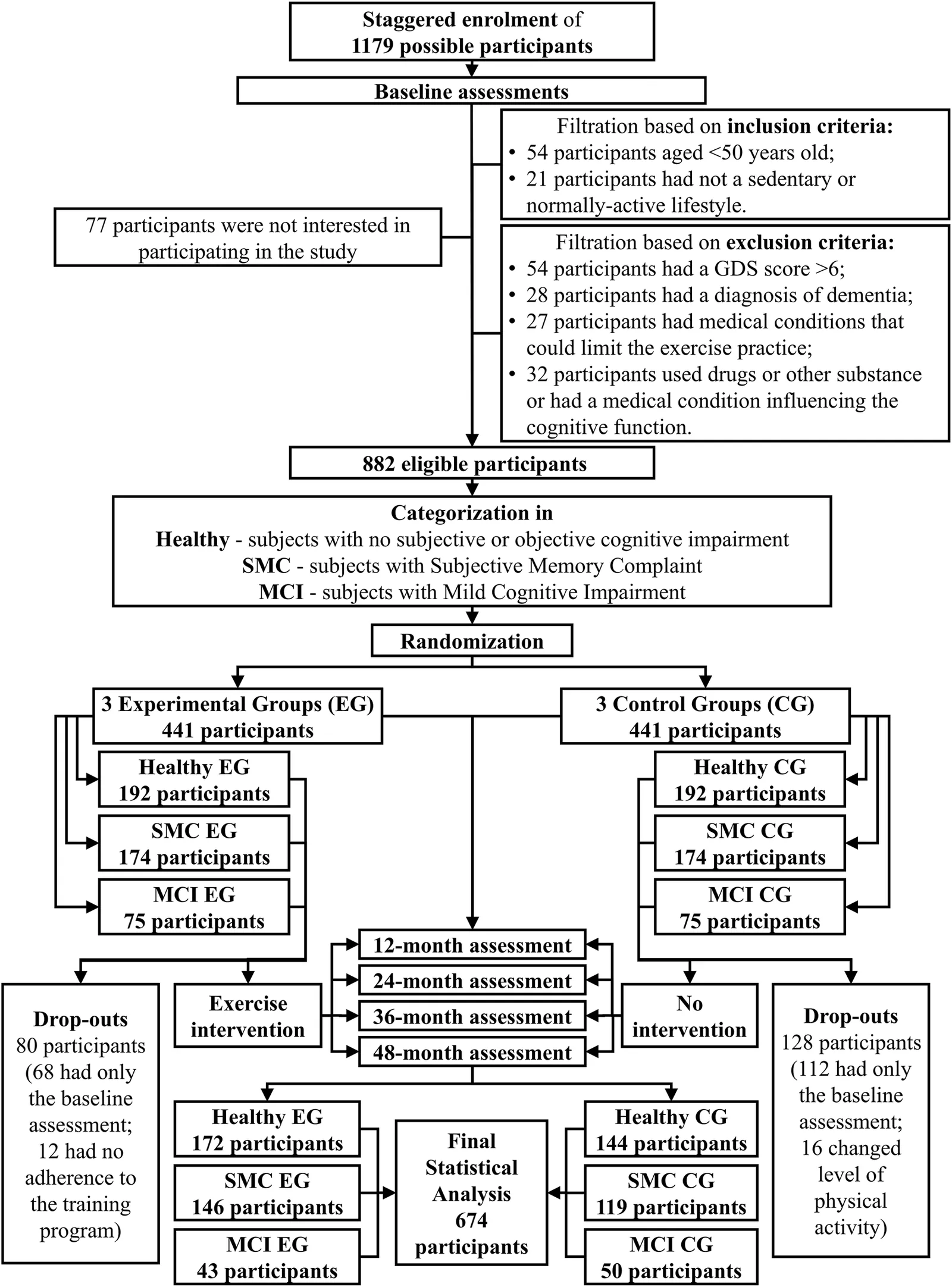
The flowchart of the study.

EPD was a parallel group, single-blind, 1:1 RCT planned following the CONSORT (http://www.consort-statement.org/) and SPIRIT (https://www.spirit-statement.org/) guidelines (Iuliano et al., 2019). The trial was registered at ClinicalTrials.gov (NCT02236416). The flowchart of the study is reported in Figure 1.

### Participants

Volunteers aged ≥50 years from community centers for older adults in Campobasso (Italy), classified as sedentary or normally active according to the definition of Pate et al. (2008), were screened for study eligibility. Exclusion criteria were as follows: (a) score of >6 on the short version of Geriatric Depression Scale (GDS); (b) alcohol intake more than 4 units/day; (c) medical conditions that could limit participation in the exercise program; (d) a diagnosis of dementia according to the International Statistical Classification of Diseases, 10th Revision (ICD-10); (e) a Mini-Mental State Examination (MMSE) score of <24; (f) a Clinical Dementia Rating (CDR) score of >1; (g) inability to walk for 6 min without assistance; and (h) use of drugs or other substances or the presence of a medical condition influencing cognitive functioning. In accordance with the recommendations proposed by the American Heart Association (Myers et al., 2009) and the (American College of Sports Medicine et al., 2010), volunteers with an intermediate or high cardiovascular risk underwent an additional ergometric test in order to evaluate their eligibility.

Participants were divided into three groups:

*Category A—healthy participants* showing normal neuropsychological performance and a Memory Complaint Questionnaire (MAC-Q) score of <25.

*Category B—participants with SMC* showing normal performance on the neuropsychological test battery and a MAC-Q score of ≥25.

*Category C—participants with MCI* meeting the diagnostic criteria of the International Working Group (Winblad et al., 2004) and having an MMSE score of ≥24, a CDR score of 0.5, and age-, sex-, and education-adjusted pathological scores on at least one neuropsychological test. The MMSE cutoff was employed because it offers a reasonable balance between sensitivity (0.85) and specificity (0.90) for detecting cognitive impairment while helping exclude individuals with overt dementia across different populations (Mitchell et al., 2009).

The study has been designed and conducted in accordance with the principles of the Declaration of Helsinki. It has been approved by the Local Ethics Committee and the Agency for Public Health of Molise Region (ASReM n. 203 of 08/03/2011). Written informed consent was obtained from all participants prior to their enrollment in the study.

### Assessments

Volunteers were invited to the Center for Research and Training in Medicine of Aging of the University of Molise for an in-person assessment that included the evaluation of health status and cognitive functioning. The neurocognitive assessment battery included MMSE, Frontal Assessment Battery (FAB), immediate and delayed recalls in the Rey’s Auditory Verbal Learning Test (RAVLT), immediate and delayed recalls in the Prose Memory Test (PMT), Attentive Matrices Test (AMT), Raven’s Colored Progressive Matrices (CPM), completion time and errors in the short version of the Stroop Word-Color Interference test (SWCT), Trail Making Test (TMT), and Copying Geometric Drawings (CGD) with and without landmarks. For all cognitive measures, higher scores indicate better performance, except for SWCT (errors and completion time) and TMT, where lower scores reflect better performance. The physical activity level of the participants was assessed using the Physical Activity Scale for the Elderly (PASE) (Covotta et al., 2018). Assessment sessions were repeated at 12, 24, 36, and 48 months.

### Blinding

The researchers involved in the assessment of the general conditions and health status, neurocognitive performances, and physical fitness level assessments, as well as the researchers involved in the data analysis, were blinded to the randomization assignment. During each follow-up assessment, participants and researchers could not provide or request information about the exercise program that participants were performing.

### Randomization

After the baseline assessment, the participants in each category group were identified by a progressive number and randomly assigned in a ratio of 1:1 to an experimental or control group, using a list of Random numbers generated by online statistical software (https://www.random.org/sequences/, Dublin, Ireland).

#### Experimental group

Participants assigned to the EG were involved in a 1-hour exercise session three times a week for 48 months. The training program was carried out at the University Sports Center of the University of Molise in small groups (ranging from 10 to 16 participants per session) by experienced personal trainers and was personalized based on physical fitness levels, health status, and exercise responses. The group format was designed to promote social interaction while ensuring sufficient individual supervision to maintain the prescribed exercise intensity.

Physical fitness parameters were assessed during the first 6 months in a single cohort of participants to validate the proposed exercise protocol by examining the initial physiological adaptations to the training program. Once the protocol was validated through significant improvements in physical performance, physical fitness tests were not repeated over time or across all subsequently recruited cohorts in order to avoid excessive physical burden on participants, as the study primarily aimed to assess cognitive improvements, which typically develop over a considerably longer timeframe. The progression of the exercise protocol was achieved through a gradual increase in exercise intensity and adjustments in recovery intervals. Exercise intensity was monitored and progressively adjusted according to Borg’s Rating of Perceived Exertion (RPE), a 6-to-20-point scale (Borg, 1974).

Each training session consisted of a warm-up, aerobic, resistance, cool-down, and stretching phase. The *warm-up phase* lasted 10 min and consisted of light-to-moderate intensity (RPE 10–12) aerobic activity, carried out through a treadmill, a stationary bike, or walking/jogging activities. The *aerobic phase* was performed for 15–20 min using a treadmill, a stationary bike, an arm-cycle ergometer and/or running. The duration and intensity of exercises were gradual over time, starting with multiple bouts (<2 min) of light-to-moderate intensity (RPE max 12–13) up to two 10-min bouts of moderate to vigorous exercise (RPE max 16–17), after several weeks of training. The *resistance phase* was performed for 20–25 min and was based on bodyweight (calisthenics) and dumbbells (up to 2 kg) exercises, involving the main muscular groups of the limbs and trunk; duration and intensity also increased progressively over time, starting with two series of 10 repetitions (RPE max 11–12) up to six series of 20 fast repetitions (RPE max 14–16) for each muscle group. The *cool-down and stretching phase*, lasting 10 min, consisted in flexibility exercises for each major muscle/tendon group. Participants were invited to stretch to the point of feeling tightness or slight discomfort, hold a static stretch for 20–30 s, and repeat the exercise two to three times for each muscle/tendon group. The training program and the RPE system were chosen to be easily reproducible at home to promote a physically active lifestyle also after the end of the study.

#### Control group

Participants assigned to this group were strongly advised to maintain their habitual sedentary lifestyle or continue their usual low-intensity PA, consisting in walking and/or stretching, toning and/or balance exercises, or postural education. Those who changed from a sedentary lifestyle to light physical activity (RPE < 11) were still included. Conversely, participants who changed their PA intensity from light to moderate or vigorous (RPE >=11) were excluded from the final statistical analysis.

#### Adherence assessment

The adherence to the PA program was computed by dividing the number of attended sessions by the number of sessions expected in 48 months (excluding preprogrammed periods of stop for holidays) and multiplying by 100. Participants who continued the study and were included in the statistical analysis demonstrated an adherence rate of at least 50% to the training sessions proposed over the 4-year period. During training sessions, to ensure high fidelity of the intervention delivered, qualitative adherence was continuously and actively managed. Experienced personal trainers monitored individual biomechanical execution and validated the prescribed intensity targets using the Borg RPE scale.

#### Adverse effects

To ensure comprehensive safety monitoring, participants were systematically asked about the presence of any adverse effects, such as musculoskeletal pain or discomfort, at each exercise session and were continuously monitored by certified trainers for acute symptoms such as angina and shortness of breath during the exercise. Additionally, participants were instructed to report any health-related incidents occurring outside the facility during their periodic evaluations. All documented adverse events were subsequently classified by the clinical staff according to the type and severity.

### Outcomes

The primary outcome was the 48-month change in MMSE scores. Secondary outcomes were the 48-month changes in neuropsychological test scores: FAB; immediate and delayed recalls of RAVLT and PMT; AMT; CPM; completion time and errors in SWCT; TMT; and CGD with and without landmarks.

### Sample size calculation

The sample size calculations were based on the findings of previous studies and were performed separately for each category group (Iuliano et al., 2019). Considering the estimated 4-year decline in the MMSE score in the three categories of participants (healthy, SMC, and MCI), the standard deviation (SD) previously reported, and the possible attrition rate and using an α level of less than 0.05, we estimated that 90 participants per arm group can guarantee a power of 0.80.

### Statistical analysis

Comparisons between the EG and CG were performed for each category A, B, and C separately. The normal distribution of the residuals was tested using the Shapiro–Wilk test and Q–Q plots. Sampling homogeneity (i.e., baseline differences between groups) was examined using the chi-square (*χ* ^2^) for categorical variables and an independent-samples t-test for normally distributed variables. Since a complete-case analysis was not feasible, as the number of participants who completed all time points represented a very small minority, data were analyzed according to an mITT approach as previously stated. Specifically, the analysis included all randomized participants who completed the baseline assessment and at least one postbaseline assessment. A linear mixed-effects model analysis with repeated measures and robust estimators (LMM) was used to evaluate significant differences among time points and between the CG and EG (fixed effect: time and group). The time*group interaction was also included in the analysis. Participants were considered a random effect. LMM was used as the main statistical analysis due to the presence of missing data in some time points. The LMM was performed under the assumption that these missing data met the missing at random condition: The analysis of the missing data mechanism for the longitudinal dataset was conducted using complementary statistical analyses, all of which converged toward a missing at random classification; the results of these analyses are reported in Supplementary Material (Paragraph 1S). Furthermore, a first-order autoregressive (AR1) or autoregressive Hilbertian of order 1 (ARH1) covariance structure was used for the analysis. Akaike information criterion (AIC) and Bayesian information criterion (BIC) of each structure were calculated, but when the indications of AIC and BIC were not concordant, model selection was guided primarily by the BIC. The data of BIC and AIC are reported in Supplementary Table S2. It is important to note that compound symmetry (CS), heterogeneous compound symmetry (CSH), and unstructured (UN) covariance structures were also tested; however, they were excluded as they resulted in a nonpositive final Hessian matrix. Consequently, only covariance structures that achieved full convergence and a positive Hessian matrix were considered.

Partial eta squared (η^2^_p_) was instead used as the measurement of the effect size of the analyses. When a significant difference was detected in the main analysis (i.e., LMM), Bonferroni correction was used to perform post-hoc pairwise comparisons between the scores at different time points and the baseline score or between the two groups for each time point. Similarly, for the omnibus analyses of the 13 neurocognitive scores, a *p*-value adjustment for multiple comparisons was used; in particular, a false discovery rate (FDR) correction was adopted using the Benjamini–Hochberg (BH) methods. Data were analyzed using SPSS v.26 (IBM Corp., Armonk, NY, United States) and R software v.4.5.2 (R Core Team, Vienna, Austria).

## Results

The baseline demographic and clinical characteristics of the participants included in the final analysis across the three diagnostic categories are reported in Table 1. The EG and CG were comparable at baseline across all three categories in terms of age, education, gender distribution, and PASE scores. The baseline characteristics of all randomized participants, as well as of those included and excluded in the final analysis, are reported in Supplementary Table S1 (Parts 1 and 2) of Supplementary Material. The Shapiro–Wilk test and the Q–Q plots indicated a non-normal distribution of the residuals.

**TABLE 1:** Baseline characteristics of the participants included in the final statistical analysis.

​	Experimental group (EG)	Control group (CG)	Both groups	Differences between EG and CG*
All participants				
Numerosity	361	313	674	-
Age (years)	63.39 ± 6.85	64.24 ± 8.00	63.78 ± 7.42	T_672_ = 1.486; *p* = 0.137
Instruction level (years)	10.94 ± 3.71	11.33 ± 4.50	11.12 ± 4.10	T_672_ = 1.233; *p* = 0.218
Gender	257 female and 104 male subjects	218 female and 95 male subjects	475 female and 199 male subjects	χ^2^ = 0.192; *p* = 0.661
PASE (score)	106.37 ± 29.73	110.44 ± 29.26	108.26 ± 29.56	T_672_ = 1.786; *p* = 0.075
Category A—healthy participants				
Numerosity	172	144	316	*-*
Age (years)	63.03 ± 7.01	63.08 ± 8.64	63.06 ± 7.78	T_314_ = 0.057; *p* = 0.955
Instruction level (years)	11.18 ± 3.58	11.78 ± 4.02	11.46 ± 3.79	T_314_ = 1.403; *p* = 0.162
Gender	115 females and 57 males	95 females and 49 males	210 females and 106 males	χ^2^ = 0.028; *p* = 0.868
PASE (score)	107.3 ± 30.4	110.57 ± 29.15	108.79 ± 29.84	T_314_ = 0.970; *p* = 0.333
Category B—participants with SMC				
Numerosity	146	119	265	*-*
Age (years)	63.45 ± 6.71	64.68 ± 7.25	64 ± 6.97	T_263_ = 1.431; *p* = 0.153
Instruction level (years)	11.10 ± 3.84	10.92 ± 4.9	11.02 ± 4.34	T_263_ = −0.335; *p* = 0.737
Gender	111 female and 35 male subjects	96 female and 23 male subjects	207 female and 58 male subjects	χ^2^ = 0.827; *p* = 0.363
PASE (score)	103.73 ± 29.27	110.45 ± 29.66	106.75 ± 29.58	T_263_ = 1.848; *p* = 0.066
Category A—participants with MCI				
Numerosity	43	50	93	*-*
Age (years)	64.6 ± 6.71	66.54 ± 7.34	65.65 ± 7.09	T_91_ = 1.322; *p* = 0.189
Instruction level (years)	10.25 ± 4.17	10.96 ± 4.79	10.25 ± 4.29	T_91_ = 0.756; *p* = 0.451
Gender	31 female and 12 male subjects	27 female and 23 male subjects	58 female and 35 male subjects	χ^2^ = 3.224; *p* = 0.072
PASE (score)	111.65 ± 28.23	110.06 ± 29.19	110.8 ± 28.6	T_91_ = −0.266; *p* = 0.791

Values are presented as means ± SDs.

SMC, subjective memory complaints; MCI, mild cognitive impairment.

* The homogeneity in baseline concerning age, instruction levels, and PASE scores between the EG and CG for the three categories was tested using a t-test; the homogeneity concerning gender distribution was instead tested using the chi-square (*χ* ^2^).

Regarding healthy participants (category A), the experimental group (EG) showed stable or improved performance across multiple cognitive domains over 48 months, whereas the control group (CG) exhibited progressive decline. Global cognitive status (MMSE) remained stable in the EG (29.37 ± 1.19 to 29.19 ± 1.23), while it declined significantly in the CG (29.40 ± 0.95 to 27.52 ± 1.34; time*group interaction: F_4,1063_ = 6.915, *p* < 0.001, η^2^_p_ = 0.025). Similarly, executive functions (FAB) improved in the EG (17.43 ± 0.97 to 17.70 ± 0.59) but declined in the CG (17.45 ± 0.89 to 17.00 ± 1.13; time*group interaction: F_4,1063_ = 3.720, *p* = 0.005, η^2^_p_ = 0.014).

The intervention yielded substantial benefits in verbal and episodic memory. For RAVLT immediate recall, the EG improved by ∼5.5 points (41.98 ± 9.44 to 47.48 ± 11.32), while the CG remained near baseline (42.85 ± 8.58 to 43.33 ± 7.54; time*group interaction: F_4,1063_ = 4.243, *p* = 0.002, η^2^_p_ = 0.016). RAVLT delayed recall improved in the EG (9.41 ± 3.04 to 10.78 ± 2.90) but declined in the CG (9.00 ± 2.39 to 8.54 ± 2.38; time*group interaction: F_4,1063_ = 6.813, *p* < 0.001, η^2^_p_ = 0.025). Prose Memory Test immediate recall also increased in the EG (6.90 ± 1.31 to 7.87 ± 0.70), while it declined in the CG (6.97 ± 1.12 to 6.77 ± 1.30; time*group interaction: F_4,1063_ = 12.972, *p* < 0.001, η^2^_p_ = 0.047), with similar patterns for delayed recall (EG: 5.79 ± 1.49 to 6.90 ± 0.99; CG: 5.86 ± 1.38 to 5.73 ± 1.27; time*group interaction: F4,1063 = 8.971, *p* < 0.001, η^2^_p_ = 0.033). Nonverbal fluid intelligence (CPM) also favored the EG (30.48 ± 4.70 to 31.47 ± 3.95 vs. CG: 29.73 ± 4.43 to 29.28 ± 4.45; time*group interaction: F_4,1063_ = 4.333, *p* = 0.002, η^2^_p_ = 0.016).

Conversely, several measures showed no significant group-by-time interactions, indicating comparable trajectories between the groups. Visual attention (AMT) showed only a main group effect (F_1,1063_ = 8.150, *p* = 0.004, η^2^_p_ = 0.008), without significant longitudinal divergence. Executive control and response inhibition (SWCT-e and SWCT-t), psychomotor speed and cognitive flexibility (TMT), and visuoconstructional abilities (CGD-wo and CGD-w) all evolved similarly in both groups, with no meaningful intervention benefits observed (all time*group interactions *p* > 0.05). Detailed results and post-hoc analyses are reported in Table 2. The F-values, the *p*-values, and the η^2^_p_ for all analyses are instead reported in Supplementary Table S3 of Supplementary Material.

**TABLE 2:** Assessment scores at baseline and 12, 24, 36, and 48 months in healthy participants.

Test		Baseline score	12-month score	24-month score	36-month score	48-month score	Significance
		Mean ± SD (n)	Mean ± SD (n)	Mean ± SD (n)	Mean ± SD (n)	Mean ± SD (n)	
MMSE	EG	29.37 ± 1.19 (172)	29.35 ± 1.16 (139)	29.28 ± 1.10 (104)	29.30 ± 1.03 (113)	29.19 ± 1.23 (79)*	Group: *p* = 0.008 Time: *p* < 0.001 Time*group: *p* < 0.001
	CG	29.40 ± 0.95 (144)	29.29 ± 1.08 (94)	29.21 ± 1.10 (85)	29.04 ± 1.15 (89)	27.52 ± 1.34 (54)#	
FAB	EG	17.43 ± 0.97 (172)	17.41 ± 1.02 (139)	17.47 ± 0.88 (104)	17.64 ± 0.66 (113)* #	17.70 ± 0.59 (79)* #	Group: *p =* 0.005 Time: *p* = 0.004 Time*group: *p* = 0.005
	CG	17.45 ± 0.89 (144)	17.32 ± 1.09 (94)	17.09 ± 1.86 (85)	17.25 ± 1.67 (89)	17.00 ± 1.13 (54)#	
RAVLT-i	EG	41.98 ± 9.44 (172)	44.57 ± 9.91 (139)#	43.94 ± 10.35 (104)* #	44.94 ± 10.21 (113)* #	47.48 ± 11.32 (79)* #	Group: *p* = 0.015 Time: *p* < 0.001 Time*group: *p* = 0.002
	CG	42.85 ± 8.58 (144)	42.66 ± 8.42 (94)	40.89 ± 7.88 (85)#	41.73 ± 7.74 (89)	43.33 ± 7.54 (54)	
RAVLT-d	EG	9.41 ± 3.04 (172)	9.75 ± 2.90 (139)*	9.10 ± 2.81 (104)*	9.73 ± 2.8 (113)* #	10.78 ± 2.90 (79)* #	Group: *p* < 0.001 Time: *p* < 0.001 Time*group: *p* < 0.001
	CG	9.00 ± 2.39 (144)	8.47 ± 2.06 (94)	8.19 ± 2.10 (85)#	8.45 ± 2.26 (89)	8.54 ± 2.38 (54)	
PMT-i	EG	6.90 ± 1.31 (172)	7.04 ± 1.21 (139)	7.40 ± 0.93 (104)* #	7.71 ± 0.72 (113)* #	7.87 ± 0.70 (79)* #	Group: *p* < 0.001 Time: *p* < 0.001 Time*group: *p* < 0.001
	CG	6.97 ± 1.12 (144)	6.75 ± 1.27 (94)	6.73 ± 1.19 (85)#	6.65 ± 1.25 (89)#	6.77 ± 1.30 (54)	
PMT-d	EG	5.79 ± 1.49 (172)	6.01 ± 1.43 (139)#	6.47 ± 1.33 (104)* #	6.74 ± 1.18 (113)* #	6.90 ± 0.99 (79)* #	Group: *p* < 0.001 Time: *p* < 0.001 Time*group: *p* < 0.001
	CG	5.86 ± 1.38 (144)	5.68 ± 1.48 (94)	5.74 ± 1.29 (85)	5.71 ± 1.28 (89)	5.73 ± 1.27 (54)	
AMT	EG	50.12 ± 5.61 (172)	50.00 ± 5.91 (139)	51.14 ± 5.14 (104)*	50.84 ± 5.47 (113)*	51.13 ± 6.53 (79)*	Group: *p* = 0.004 Time: *p* = 0.413 (NS) Time*group: *p* = 0.214 (NS)
	CG	49.17 ± 6.20 (144)	48.32 ± 6.54 (94)	48.98 ± 6.38 (85)	48.84 ± 6.29 (89)	48.48 ± 5.74 (54)	
CPM	EG	30.48 ± 4.70 (172)	29.91 ± 4.45 (139)#	29.84 ± 4.36 (104)* #	30.19 ± 4.28 (113)*	31.47 ± 3.95 (79)*	Group: *p* = 0.003 Time: *p* < 0.001 Time*group: *p* = 0.002
	CG	29.73 ± 4.43 (144)	29.69 ± 4.16 (94)	27.86 ± 4.6 (85)#	28.67 ± 4.61 (89)#	29.28 ± 4.45 (54)#	
SWCT-e	EG	0.34 ± 0.89 (172)	0.27 ± 0.77 (139)	0.24 ± 0.51 (104)	0.34 ± 0.53 (113)	0.27 ± 0.50 (79)	Group: *p* = 0.030 (NSA) Time: *p* = 0.016 Time*group: *p* = 0.522 (NS)
	CG	0.63 ± 1.72 (144)	0.56 ± 1.37 (94)#	0.36 ± 0.69 (85)	0.47 ± 1.02 (89)	0.78 ± 2.00 (54)	
SWCT-t	EG	22.86 ± 12.18 (172)	20.93 ± 10.62 (139)	20.95 ± 11.06 (104)	22.26 ± 12.23 (113)	22.09 ± 13.32 (79)	Group: *p* = 0.718 (NS) Time: *p* = 0.001 Time*group: *p* = 0.656 (NS)
	CG	24.34 ± 13.24 (144)	21.74 ± 9.96 (94)#	21.95 ± 9.07 (85)#	21.75 ± 9.47 (89)#	21.83 ± 10.83 (54)	
TMT	EG	43.55 ± 15.8 (172)	38.72 ± 12.92 (139)	39.43 ± 16.95 (104)	45.16 ± 27.4 (113)	49.44 ± 40.06 (79)	Group: *p* = 0.219 (NS) Time: *p* = 0.001 Time*group: *p* = 0.008
	CG	45.16 ± 20.71 (144)	42.57 ± 16.59 (94)	41.73 ± 18.51 (85)	44.25 ± 18.94 (89)	48.43 ± 22.13 (54)#	
CGD-wo	EG	10.38 ± 1.97 (172)	10.58 ± 1.6 (139)	10.38 ± 1.66 (104)	10.57 ± 1.59 (113)	10.58 ± 1.71 (79)	Group: *p* = 0.918 (NS) Time: *p* = 0.040 Time*group: *p* = 0.251 (NS)
	CG	10.42 ± 1.91 (144)	10.35 ± 1.6 (94)	10.32 ± 1.63 (85)	10.45 ± 1.5 (89)	10.89 ± 1.3 (54)#	
CGD-w	EG	68.43 ± 3.5 (172)	68.84 ± 2.8 (139)	68.94 ± 2.3 (104)	69.11 ± 1.75 (113)#	69.22 ± 2.02 (79)#	Group: *p* = 0.839 (NS) Time: *p* < 0.001 Time*group: *p* = 0.839 (NS)
	CG	68.65 ± 2.35 (144)	68.59 ± 2.78 (94)	68.93 ± 1.91 (85)	69.1 ± 1.46 (89)#	69.22 ± 1.41 (54)	

* = statistically significant in the comparisons with the control group (Bonferroni correction applied).

# = statistically significant in the comparisons with the baseline score (Bonferroni correction applied).

(NS) = not significant.

(NSA) = not significant after false discovery rate adjustment for multiple comparisons (the Benjamini–Hochberg method).

MMSE, Mini-Mental State Examination; FAB, Frontal Assessment Battery; RAVLT, Rey’s Auditory Verbal Learning Test; PMT, Prose Memory Test; AMT, Attentive Matrices Test; CPM, Raven’s Colored Progressive Matrices; SWCT, Stroop Word-Color Interference test; TMT, Trail Making Test; CGD, Copying Geometric Drawings.

EG, experimental group; CG, control group; -i, immediate recall; -d, delayed recall; -e, errors; -t, time; -w, with landmarks; -wo, without landmarks.

Since this is an analysis based on a modified intention-to-treat (mITT) approach, the number in parentheses represents the number of participants included in the analysis at a specific time point.

In participants with subjective memory complaints (category B), global cognitive status (MMSE) remained stable in the EG over 48 months (29.32 ± 1.42 to 29.3 ± 1.12), while it declined progressively in the CG (29.50 ± 0.96 to 27.43 ± 1.27), with a highly significant differential trajectory (time*group interaction: F_4,911_ = 7.281, *p* < 0.001, η^2^_p_ = 0.031). Executive functions (FAB), however, remained stable in both groups without meaningful divergence (time*group interaction: F_4,911_ = 1.208, *p* = 0.306, η^2^_p_ = 0.005).

The intervention generated substantial benefits in verbal and episodic memory. For RAVLT immediate recall, the EG improved by ∼6.7 points (41.16 ± 9.29 to 47.91 ± 10.29), while the CG trended downward (41.56 ± 9.33 to 40.67 ± 9.13; time*group interaction: F_4,911_ = 6.548, *p* < 0.001, η^2^_p_ = 0.028). RAVLT delayed recall increased in the EG (8.85 ± 2.97 to 10.55 ± 3.22) but decreased in the CG (9.02 ± 2.67 to 8.63 ± 3.35; time*group interaction: F_4,911_ = 4.273, *p* = 0.002, η^2^_p_ = 0.018). Similarly, Prose Memory Test immediate recall improved in the EG (6.71 ± 1.38 to 7.65 ± 0.96), while it declined in the CG (6.67 ± 1.4 to 6.52 ± 1.51; time*group interaction: F_4,911_ = 9.465, *p* < 0.001, η^2^_p_ = 0.040), with comparable patterns for delayed recall (EG: 5.76 ± 1.59 to 6.87 ± 1.05; CG: 5.66 ± 1.57 to 5.66 ± 1.40; time*group interaction: F_4,911_ = 10.798, *p* < 0.001, η^2^_p_ = 0.045). Visual attention (AMT) and nonverbal fluid reasoning (CPM) also showed strong protective effects: the EG improved (AMT: 48.09 ± 6.9 to 49.23 ± 6.53; CPM: 27.96 ± 5.56 to 29.53 ± 4.32), while the CG declined (AMT: 48.57 ± 6.13 to 45.63 ± 6.22; CPM: 28.27 ± 4.1 to 26.20 ± 5.29; both time*group interactions *p* < 0.001).

Conversely, executive control and response inhibition (SWCT-e, SWCT-t), psychomotor speed and cognitive flexibility (TMT), and visuoconstructional abilities (CGD-wo, CGD-w) tracked similarly between groups with no significant interactive advantages (all time*group interactions *p* > 0.05), indicating that these domains were unaffected by the intervention. Detailed results and post-hoc analyses are reported in Table 3. Furthermore, in this case, the F-values, the *p*-values, and the η^2^_p_ for all analyses are reported in Supplementary Table S3 of Supplementary Material.

**TABLE 3:** Assessment scores at baseline and 12, 24, 36, and 48 months in SMC participants.

Test		Baseline score	12-month score	24-month score	36-month score	48-month score	Significance
		Mean ± SD (n)	Mean ± SD (n)	Mean ± SD (n)	Mean ± SD (n)	Mean ± SD (n)	
MMSE	EG	29.32 ± 1.42 (146)	29.28 ± 1.2 (114)	29.19 ± 1.36 (97)	29.34 ± 1.20 (104)*	29.3 ± 1.12 (66)*	Group: *p* = 0.052 (NS) Time: *p* < 0.001 Time*group: *p* < 0.001
	CG	29.50 ± 0.96 (119)	29.31 ± 1.22 (80)	29.20 ± 1.17 (70)#	28.78 ± 1.26 (76)#	27.43 ± 1.27 (49)#	
FAB	EG	17.34 ± 1.33 (146)	17.43 ± 1.06 (114)	17.40 ± 1.45 (97)	17.51 ± 1.42 (104)	17.24 ± 1.07 (66)	Group: *p* = 0.257 (NS) Time: *p* = 0.025 Time*group: *p* = 0.306 (NS)
	CG	17.24 ± 1.24 (119)	17.30 ± 1.21 (80)	17.30 ± 1.33 (70)	17.22 ± 1.30 (76)	17.02 ± 1.22 (49)	
RAVLT-i	EG	41.16 ± 9.29 (146)	42.96 ± 10.45 (114)#	44.69 ± 10.28 (97)* #	46.05 ± 9.83 (104)* #	47.91 ± 10.29 (66)* #	Group: *p* = 0.003 Time: *p* < 0.001 Time*group: *p* < 0.001
	CG	41.56 ± 9.33 (119)	41.39 ± 8.96 (80)	40.34 ± 10.24 (70)	42.14 ± 10.05 (76)	40.67 ± 9.13 (49)	
RAVLT-d	EG	8.85 ± 2.97 (146)	8.93 ± 3.16 (114)	9.89 ± 3.22 (97)* #	10.40 ± 2.96 (104)* #	10.55 ± 3.22 (66)* #	Group: *p* = 0.004 Time: *p* = 0.002 Time*group: *p* = 0.002
	CG	9.02 ± 2.67 (119)	8.78 ± 2.52 (80)	8.56 ± 2.51 (70)	9.11 ± 2.61 (76)	8.63 ± 3.35 (49)	
PMT-i	EG	6.71 ± 1.38 (146)	6.65 ± 1.65 (114)	7.40 ± 0.97 (97)* #	7.62 ± 0.76 (104)* #	7.65 ± 0.96 (66)* #	Group: *p* = 0.001 Time: *p* < 0.001 Time*group: *p* < 0.001
	CG	6.67 ± 1.4 (119)	6.98 ± 1.27 (80)#	6.73 ± 1.26 (70)	6.89 ± 1.34 (76)	6.52 ± 1.51 (49)	
PMT-d	EG	5.76 ± 1.59 (146)	5.73 ± 1.62 (114)	6.41 ± 1.24 (97)* #	6.78 ± 0.99 (104)* #	6.87 ± 1.05 (66)* #	Group: *p* < 0.001 Time: *p* < 0.001 Time*group: *p* < 0.001
	CG	5.66 ± 1.57 (119)	5.96 ± 1.25 (80)#	5.79 ± 1.49 (70)	5.95 ± 1.45 (76)	5.66 ± 1.40 (49)	
AMT	EG	48.09 ± 6.9 (146)	48.93 ± 6.27 (114)	50.63 ± 5.38 (97)* #	50.40 ± 5.21 (104)* #	49.23 ± 6.53 (66)*	Group: *p* = 0.004 Time: *p* < 0.001 Time*group: *p* < 0.001
	CG	48.57 ± 6.13 (119)	48.70 ± 6.48 (80)	47.60 ± 7.02 (70)	48.00 ± 6.89 (76)	45.63 ± 6.22 (49)#	
CPM	EG	27.96 ± 5.56 (146)	28.58 ± 5.2 (114)#	28.73 ± 4.42 (97)* #	29.31 ± 4.07 (104)* #	29.53 ± 4.32 (66)* #	Group: *p* = 0.005 Time: *p* < 0.001 Time*group: *p* < 0.001
	CG	28.27 ± 4.1 (119)	28.53 ± 4.1 (80)	27.14 ± 4.54 (70)#	27.84 ± 4.56 (76)#	26.20 ± 5.29 (49)#	
SWCT-e	EG	0.57 ± 1.35 (146)	0.66 ± 1.31 (114)	0.58 ± 1.1 (97)	0.56 ± 1.1 (104)	0.67 ± 1.23 (66)	Group: *p* = 0.338 (NS) Time: *p* = 0.750 (NS) Time*group: *p* = 0.663 (NS)
	CG	0.5 ± 1.16 (119)	0.41 ± 1.06 (80)	0.61 ± 1.01 (70)	0.64 ± 0.89 (76)	0.57 ± 0.82 (49)	
SWCT-t	EG	21.28 ± 10.54 (146)	22.92 ± 10.92 (114)	21.09 ± 9.62 (97)	20.74 ± 9.2 (104)	22.58 ± 11.29 (66)	Group: *p* = 0.053 (NS) Time: *p* = 0.600 (NS) Time*group: *p* = 0.147 (NS)
	CG	23.8 ± 12.03 (119)	24.38 ± 11.28 (80)	23.26 ± 10.65 (70)	23.26 ± 10.37 (76)	23.37 ± 10.45 (49)	
TMT	EG	51.4 ± 41.31 (146)	49.1 ± 22.66 (114)	44.72 ± 20.64 (97)	45.41 ± 19 (104)	47.8 ± 21 (66)#	Group: *p* = 0.256 (NS) Time: *p* < 0.001 Time*group: *p* = 0.410 (NS)
	CG	48.85 ± 21.14 (119)	49.06 ± 21.05 (80)	47.36 ± 18.81 (70)	48.61 ± 18.49 (76)	52.65 ± 19.49 (49)#	
CGD-wo	EG	9.95 ± 2.26 (146)	9.71 ± 2.44 (114)	9.98 ± 2.12 (97)	10.28 ± 1.96 (104)	10.5 ± 1.69 (66)#	Group: *p* = 0.683 (NS) Time: *p* < 0.001 Time*group: *p* = 0.557 (NS)
	CG	9.61 ± 1.83 (119)	9.88 ± 2.31 (80)	9.8 ± 2.16 (70)	10.16 ± 2.07 (76)#	10.57 ± 1.53 (49)#	
CGD-w	EG	68.23 ± 3.5 (146)	68.49 ± 2.55 (114)	68.77 ± 3.42 (97)	68.87 ± 2.97 (104)	68.76 ± 2.69 (66)	Group: *p* = 0.809 (NS) Time: *p* = 0.001 Time*group: *p* = 0.702 (NS)
	CG	67.98 ± 2.49 (119)	68.55 ± 2.96 (80)	68.64 ± 2.19 (70)#	68.87 ± 1.94 (76)#	68.98 ± 1.64 (49)#	

* = statistically significant in the comparisons with the control group (Bonferroni correction applied).

# = statistically significant in the comparisons with the baseline score (Bonferroni correction applied).

(NS) = not significant.

(NSA) = not significant after false discovery rate adjustment for multiple comparisons (the Benjamini–Hochberg method).

SMC, subjective memory complaints; MMSE, Mini-Mental State Examination; FAB, Frontal Assessment Battery; RAVLT, Rey’s Auditory Verbal Learning Test; PMT, Prose Memory Test; AMT, Attentive Matrices Test; CPM, Raven’s Colored Progressive Matrices; SWCT, Stroop Word-Color Interference Test; TMT, Trail Making Test; CGD, Copying Geometric Drawings.

EG, experimental group; CG, control group; -i, immediate recall; -d, delayed recall; -e, errors; -t, time; -w, with landmarks; -wo, without landmarks.

Since this is an analysis based on a modified intention-to-treat (mITT) approach, the number in parentheses represents the number of participants included in the analysis at a specific time point.

In participants with MCI (category C), global cognitive status (MMSE) and executive functions (FAB) remained stable in both groups over 48 months without meaningful divergence (MMSE time*group interaction: F_4,366_ = 1.169, *p* = 0.324, η^2^_p_ = 0.013; FAB time*group interaction: F_4,366_ = 1.035, *p* = 0.389, η^2^_p_ = 0.011). Similarly, verbal memory (RAVLT immediate and delayed recall) showed sharp, parallel improvements in both groups (∼8.5 points for immediate recall; EG delayed recall: 5.23 ± 1.27 to 6.78 ± 1.34; CG delayed recall: 4.80 ± 1.50 to 6.37 ± 1.63), with no training-specific benefit (both time*group interactions *p* > 0.05).

Conversely, long-term episodic memory (Prose Memory Test) demonstrated significant interactive effects. For PMT immediate recall, the EG improved by ∼1.5 points (4.98 ± 1.33 to 6.44 ± 1.11), while the CG showed a delayed, less linear increase (5.67 ± 1.41 to 6.39 ± 1.67; time*group interaction: F_4,366_ = 4.187, *p* = 0.003, η^2^_p_ = 0.044). For PMT delayed recall, the EG improved markedly by nearly 2 points (3.88 ± 1.47 to 5.86 ± 1.23), whereas the CG displayed only a modest, late increase (4.99 ± 1.67 to 5.65 ± 1.77; time*group interaction: F_4,366_ = 7.098, *p* < 0.001, η^2^_p_ = 0.072). Visual attention (AMT) also showed a significant interaction, with the EG improving by ∼4 points (43.88 ± 8.06 to 47.84 ± 5.77) versus a smaller relative increase in the CG (46.52 ± 7.48 to 47.93 ± 4.76; time*group interaction: F_4,366_ = 5.461, *p* < 0.001, η^2^_p_ = 0.056). However, nonverbal fluid reasoning (CPM) improved similarly in both groups (EG: +2.8 points; CG: +1.1 points; time*group interaction: F_4,366_ = 1.715, *p* = 0.146, η^2^_p_ = 0.018).

Executive control, processing speed, and cognitive flexibility showed no distinct training benefits. SWCT-e interference errors remained statistically equivalent between groups (time*group interaction: F_4,366_ = 1.040, *p* = 0.386, η^2^_p_ = 0.011). Although SWCT-t showed a significant time*group interaction (F_4,366_ = 5.226, *p* < 0.001, η^2^_p_ = 0.054), this was driven by a crossover effect without overall group differences. TMT completion time evolved similarly in both groups (time*group interaction: F_4,360_ = 1.547, *p* = 0.188, η^2^_p_ = 0.017). Visuoconstructional skills showed a significant interaction only for CGD-wo (EG: 8.81 ± 2.41 to 10.06 ± 1.61; CG: 9.42 ± 2.57 to 10.13 ± 1.78; time*group interaction: F_4,366_ = 5.544, *p* < 0.001, η^2^_p_ = 0.057), whereas for CGD-w, the two groups improved in parallel (time*group interaction: F_4,366_ = 2.125, *p* = 0.077, η^2^_p_ = 0.023). Detailed results and post-hoc analyses are reported in Table 4. Furthermore, in this case, the F-values, the *p*-values, and the η^2^_p_ for all analyses are reported in Supplementary Table S3 of Supplementary Material.

**TABLE 4:** Assessment scores at baseline and 12, 24, 36, and 48 months in participants with MCI.

Test		Baseline score	12-month score	24-month score	36-month score	48-month score	Significance
		Mean ± SD (n)	Mean ± SD (n)	Mean ± SD (n)	Mean ± SD (n)	Mean ± SD (n)	
MMSE	EG	22.21 ± 1.92 (43)	22.32 ± 2.01 (34)	22.13 ± 1.83 (32)	22.3 ± 1.78 (37)	22.34 ± 1.29 (32)	Group: *p* = 0.170 (NS) Time: *p* = 0.036 Time*group: *p* = 0.324 (NS)
	CG	22.50 ± 2.03 (50)	23.26 ± 0.98 (38)	22.51 ± 1.52 (39)	22.78 ± 1.47 (41)	22.27 ± 1.51 (30)	
FAB	EG	16.84 ± 1.82 (43)	17.09 ± 0.90 (34)	17.00 ± 1.14 (32)	17.05 ± 1.45 (37)	16.66 ± 1.58 (32)	Group: *p* = 0.469 (NS) Time: *p* = 0.003 Time*group: *p* = 0.389 (NS)
	CG	16.34 ± 2.16 (50)	16.95 ± 1.87 (38)	16.56 ± 1.67 (39)	17.00 ± 1.36 (41)#	16.80 ± 1.58 (30)	
RAVLT-i	EG	25.63 ± 5.39 (43)	34.65 ± 7.77 (34)#	33.5 ± 9.41 (32)#	34.97 ± 9.13 (37)#	34.38 ± 9.3 (32)#	Group: *p* = 0.404 (NS) Time: *p* < 0.001 Time*group: *p* = 0.570 (NS)
	CG	26.1 ± 5.69 (50)	33.08 ± 8.00 (38)#	32.15 ± 8.06 (39)#	33.37 ± 8.10 (41)#	34.6 ± 8.9 (30)#	
RAVLT-d	EG	5.23 ± 1.27 (43)	6.38 ± 1.50 (34)#	6.28 ± 1.37 (32)#	6.30 ± 1.43 (37)#	6.78 ± 1.34 (32)#	Group: *p* = 0.531 (NS) Time: *p* < 0.001 Time*group: *p* = 0.531 (NS)
	CG	4.80 ± 1.50 (50)	6.50 ± 1.87 (38)#	6.26 ± 1.45 (39)#	6.32 ± 1.44 (41)#	6.37 ± 1.63 (30)#	
PMT-i	EG	4.98 ± 1.33 (43)*	5.82 ± 1.27 (34)#	6.24 ± 1.29 (32)#	6.12 ± 1.20 (37)#	6.44 ± 1.11 (32)#	Group: *p* = 0.724 (NS) Time: *p* < 0.001 Time*group: *p* = 0.003
	CG	5.67 ± 1.41 (50)	5.33 ± 1.78 (38)	5.87 ± 1.61 (39)	6.34 ± 1.75 (41)	6.39 ± 1.67 (30)	
PMT-d	EG	3.88 ± 1.47 (43)*	5.29 ± 1.36 (34)#	5.36 ± 1.36 (32)#	6.23 ± 1.35 (37)* #	5.86 ± 1.23 (32)#	Group: *p* = 0.808 (NS) Time: *p* < 0.001 Time*group: *p* < 0.001
	CG	4.99 ± 1.67 (50)	5.42 ± 2.62 (38)	5.22 ± 1.64 (39)	5.53 ± 1.63 (41)	5.65 ± 1.77 (30)	
AMT	EG	43.88 ± 8.06 (43)	43.59 ± 9.72 (34)*	46.38 ± 6.18 (32)#	47.46 ± 6.67 (37)#	47.84 ± 5.77 (32)#	Group: *p* = 0.504 (NS) Time: *p* = 0.010 Time*group: *p* < 0.001
	CG	46.52 ± 7.48 (50)	48.61 ± 5.76 (38)	45.64 ± 7.91 (39)	46.46 ± 7.47 (41)	47.93 ± 4.76 (30)	
CPM	EG	25.14 ± 6.38 (43)	25.29 ± 5.7 (34)	26.47 ± 4.17 (32)	27.49 ± 3.75 (37)#	27.91 ± 3.55 (32)#	Group: *p* = 0.568 (NS) Time: *p* < 0.001 Time*group: *p* = 0.146 (NS)
	CG	26.34 ± 6.57 (50)	27.45 ± 5.26 (38)	27.03 ± 4.67 (39)	27.49 ± 4.97 (41)#	27.50 ± 6.08 (30)	
SWCT-e	EG	1.26 ± 1.65 (43)	1.51 ± 2.02 (34)	1.17 ± 1.59 (32)	1.43 ± 1.66 (37)	1.38 ± 1.54 (32)	Group: *p* = 0.033 (NSA) Time: *p* = 0.277 (NS) Time*group: *p* = 0.386 (NS)
	CG	1.09 ± 1.43 (50)	0.64 ± 0.89 (38)	0.77 ± 1.13 (39)	0.90 ± 1.07 (41)	0.67 ± 0.83 (30)	
SWCT-t	EG	33.2 ± 15.64 (43)	27.94 ± 15.15 (34)	26.31 ± 10.16 (32)	27.24 ± 10.65 (37)	30.36 ± 11.39 (32)	Group: *p* = 0.982 (NS) Time: *p* = 0.151 (NS) Time*group: *p <* 0.001
	CG	29.21 ± 13.73 (50)	27.12 ± 21 (38)	30.45 ± 20.63 (39)	29.95 ± 18.59 (41)	25.42 ± 9.72 (30)	
TMT	EG	56.49 ± 25.06 (43)	55.91 ± 25.4 (34)#	56.16 ± 20.2 (32)	56.22 ± 19.41 (37)	56.78 ± 20.74 (32)	Group: *p* = 0.023 (NSA) Time: *p* = 0.002 Time*group: *p* = 0.188 (NS)
	CG	52.42 ± 21.03 (50)	46.39 ± 19.56 (38)	49.31 ± 26.08 (39)	52.22 ± 24.05 (41)	49.6 ± 15.08 (30)#	
CGD-wo	EG	8.81 ± 2.41 (43)	8.76 ± 2.08 (34)* #	9.75 ± 1.8 (32)#	10.14 ± 1.57 (37)* #	10.06 ± 1.61 (32)#	Group: *p* = 0.752 (NS) Time: *p* = 0.003 Time*group: *p* < 0.001
	CG	9.42 ± 2.57 (50)	10.03 ± 2.15 (38)	9.31 ± 2.37 (39)	9.41 ± 2.19 (41)	10.13 ± 1.78 (30)	
CGD-w	EG	65.88 ± 4.77 (43)	66.85 ± 3.89 (34)	68.03 ± 3.58 (32)#	68.3 ± 2.55 (37)#	68.72 ± 2.39 (32)#	Group: *p* = 0.639 (NS) Time: *p* < 0.001 Time*group: *p* = 0.077 (NS)
	CG	66.64 ± 5.99 (50)	68.32 ± 3.63 (38)	67.92 ± 3.71 (39)#	68.15 ± 3.66 (41)#	68.03 ± 3.24 (30)	

* = statistically significant in the comparisons with the control group (Bonferroni correction applied).

# = statistically significant in the comparisons with the baseline score (Bonferroni correction applied).

(NS) = not significant.

(NSA) = not significant after false discovery rate adjustment for multiple comparisons (the Benjamini–Hochberg method).

MCI, mild cognitive impairment; MMSE, Mini-Mental State Examination; FAB, Frontal Assessment Battery; RAVLT, Rey’s Auditory Verbal Learning Test; PMT, Prose Memory Test; AMT, Attentive Matrices Test; CPM, Raven’s Colored Progressive Matrices; SWCT, Stroop Word-Color Interference Test; TMT, Trail Making Test; CGD, Copying Geometric Drawings.

EG, experimental group; CG, control group; -i, immediate recall; -d, delayed recall; -e, errors; -t, time; -w, with landmarks; -wo, without landmarks.

Since this is an analysis based on a modified intention-to-treat (mITT) approach, the number in parentheses represents the number of participants included in the analysis at a specific time point.

### Adverse events

Musculoskeletal complaints, such as knee or shoulder joint discomfort, developed in 30 women and 7 men. All documented musculoskeletal symptoms resolved within 2–4 weeks of functional rest and application of nonsteroidal anti-inflammatory drugs. Three women had a fall with minor injuries. Two men reported that the episodes of lightheadedness resolved with the reduction of antihypertensive therapy. No other adverse events were reported.

## Discussion

The present RCT, which evaluated the effects of regular long-term PA on cognition in older adults, suggests that PA is an effective strategy for slowing cognitive decline and delaying the onset of dementia in both healthy participants and those with SMC, although in the latter category, the effect on global cognition, as assessed by the MMSE, only approached statistical significance.

In MCI participants, the insufficient sample size does not allow us to draw definitive conclusions. The relatively high MMSE cutoff (>24) may have led to an underidentification of eligible participants with MCI, thereby contributing to recruitment difficulties. Furthermore, the MMSE is known to have limited sensitivity for detecting mild cognitive changes, especially in highly educated individuals or those in the early stages of cognitive impairment (Mitchell et al., 2009; Arevalo-Rodriguez et al., 2021). The use of the Montreal Cognitive Assessment (MoCA) would likely have enabled us to identify a large number of individuals with MCI. However, although the MoCA is more sensitive for detecting MCI, it has been shown to have lower specificity than the MMSE, potentially leading to a higher rate of false-positive results. In contrast, the MMSE demonstrates greater specificity for identifying more advanced cognitive impairment and overt dementia, making it a more appropriate tool when the primary aim is to exclude individuals with clear dementia (Mitchell et al., 2009; Arevalo-Rodriguez et al., 2021; Freitas et al., 2013; Nasreddine et al., 2005). Nevertheless, even with the smaller sample, the findings suggest that the intervention is less effective in individuals with MCI. Current evidence indicates that while PA is effective in reducing the risk of developing dementia and may have beneficial effects in earlier stages, such as MCI, its impact on cognitive outcomes once dementia is established appears limited. Several systematic reviews and randomized controlled trials have reported inconsistent or nonsignificant effects of exercise on cognitive function in individuals with dementia, despite some benefits in physical function and activities of daily living (Lu et al., 2026; Sun et al., 2025; Forbes et al., 2015; Lamb et al., 2018). These findings suggest that once the neurodegenerative process is clinically manifested, exercise is unlikely to substantially modify disease progression, although it may still provide important supportive and functional benefits. We hypothesize that despite the use of the MMSE, several enrolled participants may already have been in the early stages of dementia.

In healthy participants and those with SMC, global cognitive functioning, as assessed by the MMSE (primary outcome), remained essentially stable over 48 months in the EG but declined significantly in the CG. Furthermore, memory performance, as assessed by RAVLT and PMT; reasoning abilities, as assessed by CPM; and selective and sustained attention, as assessed by AMT, were significantly better in the EG than in the CG in both healthy participants and those with SMC (Tables 3, 4).

In the Lifestyle Interventions and Independence for Elders (LIFE) study, which enrolled 1,635 community-dwelling participants over 24 months of follow-up, a moderate-intensity physical activity program, compared with a health education program, did not result in improvements in global or domain-specific cognitive function (Sink et al., 2015). Apart from the shorter duration of the intervention, the greater average age of the participants and the health education intervention in the control group might explain the different results compared with our study. In the LIFE study, the mean + SD age of participants was 78.9 + 5.2 years, while in our study, it was 63.8 + 7.4 years. Although the optimal timing for implementing an active lifestyle remains unclear (Montero Odasso et al., 2020), most studies indicate that implementing an active lifestyle during childhood or middle age can mitigate cognitive decline in old age (Soldan et al., 2021). Therefore, the earlier an individual adopts an active lifestyle, the greater the cognitive benefits they are likely to achieve. Furthermore, the health education intervention might have reduced other risk factors for dementia in the control group and therefore might have reduced cognitive decline (Livingston et al., 2020).

In the Generation 100 study, which enrolled 945 community-dwelling participants over 5 years of follow-up, a moderate- or high-intensity aerobic exercise program compared with the Norwegian Health Authorities’ recommendations for physical activity did not result in improvements in cognitive function (Zotcheva et al., 2022). The greater average age of the participants and the type of intervention in the experimental and control groups might explain the different results compared with our study. In the Generation 100 study, the mean + SD age of participants was 78.2 + 2.0 years, while in our study, it was 63.8 + 7.4 years. Again, older age can explain the lack of effect on cognitive function. Regarding the type of exercise to practice, the majority of research has focused on aerobic exercise showing a promising effect on several cognitive and noncognitive outcomes (Venegas-et al., 2022). However, resistance exercise may also enhance cognitive outcomes in older adults (Northey et al., 2018; Turner et al., 2021), with superior effects over other exercise modalities (Gallardo-Gómez et al., 2022). The World Health Organization (WHO) recommends that older adults engage in 150–300 min of moderate-intensity aerobic physical activity per week; or 75–150 min of vigorous-intensity aerobic physical activity; and, at least, two times a week of muscle-strengthening activities involving major muscle groups (Bull et al., 2020). Emerging literature also highlights the effectiveness of multicomponent exercise (combining aerobic, endurance, balance, and flexibility exercises) to improve the global cognition of older adults (Yan et al., 2023). We hypothesize that the positive effect of our intervention protocol is linked to the combination of aerobic, resistance, and flexibility exercises, compared with the Zotcheva et al. (2022) protocol, which included only aerobic exercises. Furthermore, the CG participants in the Zotcheva et al. (2022) study followed the Norwegian Health Authorities’ recommendation of 30 min of moderate-intensity PA almost every day. In our study, CG participants were sedentary or engaged in low-intensity PA, and those who changed their PA intensity from light to moderate or vigorous were excluded from the final statistical analysis. Again, we speculate that the different PA protocols in the CG may explain the lack of effect in the Generation 100 study.

In the DR’s EXTRA study, which enrolled 1,410 community-dwelling participants over 4 years of follow-up, resistance exercise, aerobic exercise, diet, combined resistance exercise and diet, and combined aerobic exercise and diet programs were compared with general recommendations on physical activity and diet. The study showed only a trend toward improved cognition over 4 years in the combined aerobic exercise and diet group compared with the control group and no effect of either of these interventions alone, resistance training alone, or resistance exercise with a healthy diet on cognition (Komulainen et al., 2021). As acknowledged by the authors themselves, the lack of significant effects of aerobic or resistance exercise might be due to the fact that the cohort recruited was already physically active at baseline, the exercises were performed without supervision and without being combined, and individuals in the control group may also have improved their lifestyle during the study.

### Strengths

To our knowledge, the EPD study is the third largest and longest RCT of a PA intervention in older adults, normal or at increased risk for dementia, after the Generation 100 and DR’s EXTRA studies. Other strengths include the detailed protocol training in the EG, which is designed to be easily implemented in a real-world scenario and to encourage a healthy lifestyle in the elderly, and the comprehensive standardized, well-validated cognitive assessments. This study provides a motor training program that requires no specialized equipment for either administration or monitoring. Even if the success of the intervention heavily relies on the instructor’s role in ensuring proper execution and participant safety, the group-based nature of the activity significantly mitigates individual expenses. By adopting a group format rather than a one-on-one training model, the program effectively reduces costs, making professional supervision more sustainable. Additionally, as participants gain technical autonomy and conditioning, the low complexity of the exercises allows for a transition toward independent, home-based training, further enhancing the program’s long-term sustainability. In effect, it can be easily performed in a home setting with minimal supervision and could potentially be utilized within a tele-exercise framework. Tele-exercise programs have recently been demonstrated as effective and scalable alternatives to maintain functional health while reducing the economic burden on healthcare systems (Iuliano et al., 2025; Della Valle et al., 2025; Cantoia et al., 2025). Furthermore, the proposed activity is a multicomponent program that addresses both cardiorespiratory fitness and muscular strength. This is fully aligned with the physical activity guidelines for older adults recommended by the World Health Organization (WHO) (Bull et al., 2020).

This study also contributes to the literature by clarifying why previous trials found no cognitive benefits of physical activity and under which conditions such benefits may emerge. First, it highlights the importance of earlier intervention, showing that participants were younger than in prior studies (e.g., LIFE and Generation 100), suggesting that adopting an active lifestyle in midlife is more effective for preserving cognition. Second, it emphasizes the role of the control group: unlike previous studies where controls received health education or were already active, this study used a more sedentary comparison group, making effects easier to detect. Third, it suggests that multicomponent exercise programs (aerobic, resistance, and flexibility) may be more beneficial than single-modality interventions, which were common in earlier trials. Finally, it points to the importance of structured and supervised interventions, as opposed to less controlled or unsupervised programs. Overall, the study helps explain inconsistent findings in the literature and suggests that age, intervention design, and control group characteristics are the key factors influencing cognitive outcomes.

### Limitations

A limitation of the present study is the use of the MMSE as the primary measure of global cognitive functioning. As expected in populations without objective cognitive impairment, participants in both the healthy and SMC groups showed a clear ceiling effect at baseline, with mean scores exceeding 29 out of 30. Another limitation is that physical tests were not repeated across all cohorts to minimize participant load, given that the primary aim of the study was to assess cognitive improvements. Furthermore, the RPE scale used to measure the PA intensity had both advantages and drawbacks. On the one hand, it facilitated easier management of training sessions; on the other hand, it posed challenges in accurately identifying the effective metabolic load of the sessions. Consequently, it is not possible to state that all participants trained at the same intensity throughout the study. Another limitation of the EPD study is the high number of dropouts, a problem common to all studies with a long follow-up duration, especially if older adults are included. In particular, given that few participants underwent all the assessments (at baseline and at 12, 24, 36, and 48 months), it was not possible to carry out a complete-case statistical analysis. We addressed the issue using the LMM analysis that can handle missing values without excluding entire cases from the analysis and has superior statistical power as well as less stringent assumptions compared with other statistical approaches such as repeated measures analysis of covariance (Hilbert et al., 2019). Nevertheless, the presence of missing data at various time points may still introduce bias. Moreover, the absence of direct measures for assessing physical activity in both groups, such as accelerometers or heart rate monitors, may represent a limitation of this study. However, this choice was motivated by the desire to maintain a simple protocol that would be easily reproducible, even after the conclusion of the study project. A further limitation of this study is that adverse events were systematically collected only in the experimental group, as the primary focus was to monitor the safety of the exercise intervention. As a result, we were unable to compare the incidence of adverse events between groups, preventing direct comparisons between groups and limiting a comprehensive evaluation of safety outcomes. Lastly, as previously mentioned, the data obtained from the MCI group must be interpreted with great caution, as statistical power and adequate sample size were not achieved. Furthermore, within the same MCI group, as shown in Table 4, the EG and CG exhibited a baseline difference regarding the PMT; therefore, the results of this test must be interpreted with additional caution.

## Conclusion

In community-dwelling older adults, normal or at risk of dementia, with a sedentary or normally active lifestyle, 48 months of a multicomponent exercise program was associated with significant beneficial effects on several cognitive performances. The program produced an improvement of memory performance, reasoning abilities, and selective and sustained attention; global cognitive function remained essentially stable over 48 months in the EG, while it significantly declined in the CG. These findings suggest that PA could be an effective tool for slowing cognitive decline and delaying the onset of dementia in healthy and SMC participants.

## Data Availability

The raw data supporting the conclusions of this article will be made available by the authors without undue reservation.

## Group members of the EPD Study Group

Giovanna Aquino, Vittorio Arcari, Lorenzo Buongusto, Giuseppina Cavallo, Adriana Cristofano, Rita D’Aversa, Andrea Buonsenso, Mario Faraone, Nicola Ferrara, Mariella Filangieri, Melissa Fiscarelli, Sandro Iavarone, Francesca Iannetta, Stefano Moffa, Pasquale Mignogna, Giovannangelo Oriani, Federica Palombo, Tina Panichella, Silvia Pedata, Bruno Petti, Marilinda Spaziano, Maurizio Taglialatela, and Roberto Valente.

## Glossary

AMT: Attentive Matrices Test
CDR: Clinical Dementia Rating
CG: Control Group
CGD: Copying Geometric Drawings
CPM: Raven’s Colored Progressive Matrices
-d: Delayed Recall
-e: Errors
EG: Experimental group
EPD: Physical Exercise for Prevention of Dementia
FAB: Frontal Assessment Battery
GDS: Geriatric Depression Scale
-i: Immediate recall
ICD-10: International Statistical Classification of Diseases-10th Revision
LIFE: Lifestyle Interventions and Independence for Elders
LMM: Linear Mixed-Effects Models with Repeated Measures
MAC-Q: Memory Complaint Questionnaire
MCI: Mild Cognitive Impairment
MMSE: Mini-Mental State Examination
PA: Physical Activity
PMT: Prose Memory Test
RAVLT: Rey’s Auditory Verbal Learning Test
RCT: Randomized Controlled Trial
RPE: Borg’s Rating of Perceived Exertion
SMC: Subjective Memory Complaint
SWCT: Stroop Word-Color Interference test
-t: Time
TMT: Trail Making Test
-w: With landmarks
WHO: World Health Organization
-wo: Without landmarks
Sec: Seconds
Cm: Centimeter
SD: Standard deviation
Reps: Repetitions

## References

[B42] American College of Sports Medicine (2010). ACSM’s guidelines for exercise testing and prescription. Editor Thompson W. R. Gordon N. F. Pescatello L. S. 8th ed. Lippincott Williams & Wilkins. 10.1249/JSR.0b013e31829a68cf 23851406

[B1] Arevalo-Rodriguez I. Smailagic N. Roqué-Figuls M. Ciapponi A. Sanchez-Perez E. Giannakou A. (2021). Mini-Mental State Examination (MMSE) for the early detection of dementia in people with mild cognitive impairment (MCI). Cochrane Database Syst. Rev. 7 (7), CD010783. 10.1002/14651858.CD010783.pub3 34313331 PMC8406467

[B2] Borg G. A. (1974). Perceived exertion. Exerc Sport Sci. Rev. 2, 131–153. 4466663

[B3] Bull F. C. Al-Ansari S. S. Biddle S. Borodulin K. Buman M. P. Cardon G. (2020). World health organization 2020 guidelines on physical activity and sedentary behaviour. Br. J. Sports Med. 54 (24), 1451–1462. 10.1136/bjsports-2020-102955 33239350 PMC7719906

[B4] Cantoia M. Zimatore G. Matteo B. Sausa M. Fabbrizio A. De Giorgio A. (2025). Physical and psychological optimization of tele-exercise programs by establishing guidelines. Sci. Rep. 15 (1), 26878. 10.1038/s41598-025-10697-5 40707544 PMC12290079

[B5] Chen R. Zhao B. Huang J. Zhang M. Wang Y. Fu J. (2024). The effects of different exercise interventions on patients with subjective cognitive decline: a systematic review and network meta-analysis. J. Prev. Alzheimers Dis. 11 (3), 620–631. 10.14283/jpad.2024.65 38706278 PMC11060994

[B6] Ciria L. F. Román-Caballero R. Vadillo M. A. Holgado D. Luque-Casado A. Perakakis P. (2023). An umbrella review of randomized control trials on the effects of physical exercise on cognition. Nat. Hum. Behav. 7 (6), 928–941. 10.1038/s41562-023-01554-4 36973359

[B7] Covotta A. Gagliardi M. Berardi A. Maggi G. Pierelli F. Mollica R. (2018). Physical activity scale for the elderly: translation, cultural adaptation, and validation of the Italian version. Curr. Gerontol. Geriatr. Res. 2018, 1–7. 10.1155/2018/8294568 30224917 PMC6129314

[B8] Cummings J. Zhou Y. Lee G. Zhong K. Fonseca J. Cheng F. (2023). Alzheimer’s disease drug development pipeline: 2023. Alzheimers Dement. Transl. Res. Clin. Interv. 9 (2), e12385. 10.1002/trc2.12385 37251912 PMC10210334

[B9] Della Valle C. Gatti C. Bricca A. Mancini V. Qipo O. Masiulis N. (2025). Effects of digital physical activity interventions on muscle mechanical function in community-dwelling older adults: a systematic review and meta-analysis. Eur. Rev. Aging Phys. Act. 22 (1), 14. 10.1186/s11556-025-00380-z 40898014 PMC12403258

[B10] Demurtas J. Schoene D. Torbahn G. Marengoni A. Grande G. Zou L. (2020). Physical activity and exercise in mild cognitive impairment and dementia: an umbrella review of intervention and observational studies. J. Am. Med. Dir. Assoc. 21 (10), 1415–1422.e6. 10.1016/j.jamda.2020.08.031 32981668

[B11] Flier W. M. van der Vugt M. E. de Smets E. M. A. Blom M. Teunissen C. E. (2023). Towards a future where Alzheimer’s disease pathology is stopped before the onset of dementia. Nat. Aging 3 (5), 494–505. 10.1038/s43587-023-00404-2 37202515

[B12] Forbes D. Forbes S. C. Blake C. M. Thiessen E. J. Forbes S. (2015). Exercise programs for people with dementia. Cochrane Database Syst. Rev. 2015 (4). CD006489. 10.1002/14651858.CD006489.pub4 25874613 PMC9426996

[B13] Freitas S. Simões M. R. Alves L. Santana I. (2013). Montreal cognitive assessment: validation study for mild cognitive impairment and alzheimer disease. Alzheimer Dis. Assoc. Disord. 27 (1), 37–43. 10.1097/WAD.0b013e3182420bfe 22193353

[B14] Gallardo-Gómez D. Pozo-Cruz J. Noetel M. Álvarez-Barbosa F. Alfonso-Rosa R. M. Cruz B. del P. (2022). Optimal dose and type of exercise to improve cognitive function in older adults: a systematic review and bayesian model-based network meta-analysis of RCTs. Ageing Res. Rev. 76, 101591. 10.1016/j.arr.2022.101591 35182742

[B15] Hilbert S. Stadler M. Lindl A. Naumann F. Buhner M. (2019). Analyzing longitudinal intervention studies with linear mixed models. TPM - Test. Psychom. Methodol. Appl. Psychol. 26, 101–119. 10.4473/TPM26.1.6

[B16] Iuliano E. Cagno A. di Cristofano A. Angiolillo A. D'Aversa R. Ciccotelli S. (2019). Physical exercise for prevention of dementia (EPD) study: background, design and methods. BMC Public Health 19 (1), 659. 10.1186/s12889-019-7027-3 31142290 PMC6542067

[B17] Iuliano E. Zimatore G. Fabbrizio A. De Giorgio A. Sausa M. Matteo B. M. (2025). Tele-exercise for fitness: physical and psychological outcomes in athletes and non-athletes’ trainees. Healthcare 13 (4), 354. 10.3390/healthcare13040354 39997229 PMC11855133

[B18] Kaufman M. Dyrek P. Fredericson M. Oppezzo M. Roche M. Frehlich L. (2024). The role of physical exercise in cognitive preservation: a systematic review. Am. J. Lifestyle Med. 18 (4), 574–591. 10.1177/15598276231201555 39262880 PMC11384842

[B19] Komulainen P. Tuomilehto J. Savonen K. Männikkö R. Hassinen M. Lakka T. A. (2021). Exercise, diet, and cognition in a 4-year randomized controlled trial: dose-responses to exercise training (DR’s EXTRA). Am. J. Clin. Nutr. 113 (6), 1428–1439. 10.1093/ajcn/nqab018 33742194 PMC8244125

[B20] Kumaran K. R. Yunusa S. Perimal E. Wahab H. Müller C. P. Hassan Z. (2023). Insights into the pathophysiology of alzheimer’s disease and potential therapeutic targets: a current perspective. J. Alzheimers Dis. 91 (2), 507–530. 10.3233/JAD-220666 36502321

[B21] Lamb S. E. Sheehan B. Atherton N. Nichols V. Collins H. Mistry D. (2018). Dementia and physical activity (DAPA) trial of moderate to high intensity exercise training for people with dementia: randomised controlled trial. BMJ 16, k1675. 10.1136/bmj.k1675 PMC5953238 29769247

[B22] Liu L. Dong H. Jin X. Brooke-Wavell K. (2022). Tackling dementia: a systematic review of interventions based on physical activity. J. Geriatr. Phys. Ther. 45 (4), E169–E180. 10.1519/JPT.0000000000000332 34570041

[B23] Livingston G. Huntley J. Sommerlad A. Ames D. Ballard C. Banerjee S. (2020). Dementia prevention, intervention, and care: 2020 report of the Lancet commission. Lancet 396 (10248), 413–446. 10.1016/S0140-6736(20)30367-6 32738937 PMC7392084

[B24] López-Ortiz S. Lista S. Valenzuela P. L. Pinto-Fraga J. Carmona R. Caraci F. (2023). Effects of physical activity and exercise interventions on Alzheimer’s disease: an umbrella review of existing meta-analyses. J. Neurol. 270 (2), 711–725. 10.1007/s00415-022-11454-8 36342524

[B25] Lu C. W. Ng T. C. Cheng Y. C. Su C. H. (2026). Exercise interventions for cognitive and functional outcomes in dementia: a systematic review and meta-analysis exploring dose metrics, heterogeneity, and implementation-relevant factors. Healthcare 14 (5), 689. 10.3390/healthcare14050689 41827641 PMC12985021

[B26] Mitchell A. J. Shiri‐Feshki M. (2009). Rate of progression of mild cognitive impairment to dementia – meta‐analysis of 41 robust inception cohort studies. Acta Psychiatr. Scand. 119 (4), 252–265. 10.1111/j.1600-0447.2008.01326.x 19236314

[B27] Montero Odasso M. Ismail Z. Livingston G. (2020). One third of dementia cases can be prevented within the next 25 years by tackling risk factors. The case “for” and “against.”. Alzheimers Res. Ther. 12 (1), 81. 10.1186/s13195-020-00646-x 32641088 PMC7346354

[B28] Morton K. Heindl B. Clarkson S. Bittner V. (2022). Primordial prevention of atherosclerotic cardiovascular disease. J. Cardiopulm. Rehabil. Prev. 42 (6), 389–396. 10.1097/HCR.0000000000000748 36342681

[B29] Myers J. Arena R. Franklin B. Pina I. Kraus W. E. McInnis K. (2009). Recommendations for clinical exercise laboratories. Circulation 119 (24), 3144–3161. 10.1161/CIRCULATIONAHA.109.192520 19487589

[B30] Nasreddine Z. S. Phillips N. A. Bédirian V. Charbonneau S. Whitehead V. Collin I. (2005). The Montreal cognitive assessment, MoCA: a brief screening tool for mild cognitive impairment. J. Am. Geriatr. Soc. 53 (4), 695–699. 10.1111/j.1532-5415.2005.53221.x 15817019

[B31] Nichols E. Steinmetz J. D. Vollset S. E. Fukutaki K. Chalek J. Abd-Allah F. (2022). Estimation of the global prevalence of dementia in 2019 and forecasted prevalence in 2050: an analysis for the global burden of disease study 2019. Lancet Public Health 7 (2), e105–e125. 10.1016/S2468-2667(21)00249-8 34998485 PMC8810394

[B32] Northey J. M. Cherbuin N. Pumpa K. L. Smee D. J. Rattray B. (2018). Exercise interventions for cognitive function in adults older than 50: a systematic review with meta-analysis. Br. J. Sports Med. 52 (3), 154–160. 10.1136/bjsports-2016-096587 28438770

[B33] Pate R. R. O’Neill J. R. Lobelo F. (2008). The evolving definition of “sedentary.”. Exerc Sport Sci. Rev. 36 (4), 173–178. 10.1097/JES.0b013e3181877d1a 18815485

[B34] Rao R. V. Subramaniam K. G. Gregory J. Bredesen A. L. Coward C. Okada S. (2023). Rationale for a multi-factorial approach for the reversal of cognitive decline in alzheimer’s disease and MCI: a review. Int. J. Mol. Sci. 24 (2), 1659. 10.3390/ijms24021659 36675177 PMC9865291

[B35] Rookes T. A. Frost R. Marston L. Armstrong M. Barrado-Martin Y. Walters K. (2026). Evidence for health promotion interventions to improve cognitive and physical functioning outcomes in older adults with MCI: a state-of-the-art review. Arch. Gerontol. Geriatr. 140, 106049. 10.1016/j.archger.2025.106049 41056697

[B36] Sink K. M. Espeland M. A. Castro C. M. Church T. Cohen R. Dodson J. A. (2015). Effect of a 24-Month physical activity intervention vs health education on cognitive outcomes in sedentary older adults. JAMA 314 (8), 781–790. 10.1001/jama.2015.9617 26305648 PMC4698980

[B37] Soldan A. Pettigrew C. Zhu Y. Wang M. C. Bilgel M. Hou X. (2021). Association of lifestyle activities with functional brain connectivity and relationship to cognitive decline among older adults. Cereb. Cortex 31 (12), 5637–5651. 10.1093/cercor/bhab187 34184058 PMC8568002

[B38] Steichele K. Keefer A. Dietzel N. Graessel E. Prokosch H. U. Kolominsky-Rabas P. L. (2022). The effects of exercise programs on cognition, activities of daily living, and neuropsychiatric symptoms in community-dwelling people with dementia—a systematic review. Alzheimers Res. Ther. 14 (1), 97. 10.1186/s13195-022-01040-5 35869496 PMC9306176

[B39] Sun G. Ding X. Zheng Z. Ma H. (2025). Effects of exercise interventions on cognitive function in patients with cognitive dysfunction: an umbrella review of meta-analyses. Front. Aging Neurosci. 17, 1553868. 10.3389/fnagi.2025.1553868 40454205 PMC12122535

[B40] Turner D. T. Hu M. X. Generaal E. Bos D. Ikram M. K. Heshmatollah A. (2021). Physical exercise interventions targeting cognitive functioning and the cognitive domains in nondementia samples: a systematic review of meta-analyses. J. Geriatr. Psychiatry Neurol. 34 (2), 91–101. 10.1177/0891988720915523 32295450 PMC7859677

[B41] Venegas-Sanabria L. C. Cavero-Redondo I. Martínez-Vizcaino V. Cano-Gutierrez C. A. Álvarez-Bueno C. (2022). Effect of multicomponent exercise in cognitive impairment: a systematic review and meta-analysis. BMC Geriatr. 22 (1), 617. 10.1186/s12877-022-03302-1 35879665 PMC9316334

[B43] Winblad B. Palmer K. Kivipelto M. Jelic V. Fratiglioni L. Wahlund L. O. (2004). Mild cognitive impairment – beyond controversies, towards a consensus: report of the international working group on mild cognitive impairment. J. Intern. Med. 256 (3), 240–246. 10.1111/j.1365-2796.2004.01380.x 15324367

[B44] Yan J. Li X. Guo X. Lin Y. Wang S. Cao Y. (2023). Effect of multicomponent exercise on cognition, physical function and activities of daily life in older adults with dementia or mild cognitive impairment: a systematic review and meta-analysis. Arch. Phys. Med. Rehabil. 104 (12), 2092–2108. 10.1016/j.apmr.2023.04.011 37142178

[B45] Zotcheva E. Håberg A. K. Wisløff U. Salvesen Ø. Selbæk G. Stensvold D. (2022). Effects of 5 years aerobic exercise on cognition in older adults: the generation 100 study: a randomized controlled trial. Sports Med. 52 (7), 1689–1699. 10.1007/s40279-021-01608-5 34878637 PMC9213353

