# Supplementary material for: Physical exercise for prevention of dementia study: a 48-month randomized controlled trial
Source: Front Aging. 2026 Aug 10;7:1847451. doi: 10.3389/fragi.2026.1847451 (PMC13501581; doi:10.3389/fragi.2026.1847451)
Supplement: Supplementary file 1 [file Supplementaryfile1.pdf]

Table 1S – Part 1. Baseline characteristics of participants included in the final statistical analysis versus those randomized

	Experimental groups (EG)			Control groups (CG)		
	**Randomized**	**Analysed**	**Statistical diff.***	**Randomized**	**Analysed**	**Statistical diff.***
**Category A - Healthy participants**						
Numerosity	192	172	-	192	144	-
Age (years)	62.43 ± 7.34	63.03 ± 7.01	T_{362}=1.233; p=0.427	63.22 ± 10.38	63.08 ± 8.64	T_{334}=-0.131; p=0.896
Instruction level (years)	11.13 ± 3.66	11.18 ± 3.58	T_{362}=0.131; p=0.895	11.44 ± 4.00	11.78 ± 4.02	T_{334}=0.769; p=0.442
Gender	130 f and 62 m	115 f and 57 m	χ^2= 0.030; p=0.863	127 f and 65 m	95 f and 49 m	χ^2= 0.001; p=0.973
PASE (score)	108.09 ± 30.16	107.3 ± 30.4	T_{362}=-0.249; p=0.804	113.43 ± 35.33	110.57 ± 29.15	T_{334}=-0.790; p=0.430
**Category B - Participants with SMC**						
Numerosity	174	146	-	174	119	-
Age (years)	63.83 ± 6.81	63.45 ± 6.71	T_{318}=-0.501; p=0.617	65.08 ± 7.33	64.68 ± 7.25	T_{291}=-0.461; p=0.645
Instruction level (years)	11.16 ± 3.91	11.10 ± 3.84	T_{318}=-0.138; p=0.890	10.51 ± 4.79	10.92 ± 4.90	T_{291}=0.713; p=0.476
Gender	132 f and 42 m	111 f and 35 m	χ^2= 0.001; p=0.972	139 f and 35 m	96 f and 23 m	χ^2= 0.028; p=0.868
PASE (score)	104.69 ± 29.58	103.73 ± 29.27	T_{318}=-0.291; p=0.772	112.45 ± 36.32	110.45 ± 29.66	T_{291}=-0.498; p=0.619
**Category C - Participants with MCI**						
Numerosity	75	43	-	75	50	-
Age (years)	64.81 ± 6.33	64.6 ± 6.71	T_{116}=-0.170; p=0.866	66.75 ± 7.18	66.54 ± 7.34	T_{123}=-0.159; p=0.874
Instruction level (years)	10.8 ± 3.92	10.25 ± 4.17	T_{116}=-0.717; p=0.475	10.49 ± 4.78	10.96 ± 4.79	T_{123}=0.538; p=0.591
Gender	55 f and 20 m	31 f and 12 m	χ^2= 0.021; p=0.884	46 f and 29 m	27 f and 23 m	χ^2= 0.664; p=0.415
PASE (score)	112.16 ± 29.27	111.65 ± 28.23	T_{116}=-0.092; p=0.927	112.51 ± 30.87	110.06 ± 29.19	T_{123}=-0.444; p=0.658

Values are means ± SDs

SMC = subjective memory complaints; MCI = mild cognitive impairment;

* the comparisons in baseline concerning age, instruction levels and PASE scores between EG and CG for the 3 categories, were tested using T-test; the homogeneity concerning gender distribution was instead tested using chi-square (χ^2).

Table 1S – Part 2. Baseline characteristics of participants included in the final statistical analysis versus those not included**.**

	Experimental groups (EG)			Control groups (CG)		
	**Not analysed**	**Analysed**	**Statistical diff.***	**Not analysed**	**Analysed**	**Statistical diff.***
**Category A - Healthy participants**						
Numerosity	20	172	-	48	144	-
Age (years)	57.25 ± 8.23	63.03 ± 7.01	T_{190}=3.426; *p*<0.001	63.65 ± 14.51	63.08 ± 8.64	T_{190}=-0.329; *p*=0.743
Instruction level (years)	10.7 ± 4.55	11.18 ± 3.58	T_{190}=0.551; *p*=0.582	10.8 ± 3.79	11.78 ± 4.02	T_{190}=1.483; *p*=0.140
Gender	15 f and 5 m	115 f and 57 m	χ^2= 0.543; *p*=0.461	32 f and 16 m	95 f and 49 m	χ^2= 0.008; *p*=0.930
PASE (score)	114.9 ± 27.76	107.3 ± 30.4	T_{190}=-1.067; *p*=0.287	121.00 ± 48.02	110.57 ± 29.15	T_{190}=-1.799; *p*=0.074
**Category B - Participants with SMC**						
Numerosity	28	146	-	55	119	-
Age (years)	65.82 ± 7.09	63.45 ± 6.71	T_{172}=-1.697; *p*=0.092	65.95 ± 7.49	64.68 ± 7.25	T_{172}=-1.063; *p*=0.289
Instruction level (years)	11.46 ± 4.32	11.10 ± 3.84	T_{172}=-0.445; *p*=0.657	9.62 ± 4.45	10.92 ± 4.90	T_{172}=1.674; *p*=0.096
Gender	21 f and 7 m	111 f and 35 m	χ^2= 0.013; *p*=0.907	43 f and 12 m	96 f and 23 m	χ^2= 0. 145; *p*=0.907
PASE (score)	109.68 ± 31.23	103.73 ± 29.27	T_{172}=-0.975; *p*=0.331	116.76 ± 47.72	110.45 ± 29.66	T_{172}=-1.066; *p*=0.703
**Category C - Participants with MCI**						
Numerosity	32	43	-	25	50	-
Age (years)	65.09 ± 5.87	64.6 ± 6.71	T_{73}=-0.330; *p*=0.745	67.16 ± 6.99	66.54 ± 7.34	T_{73}=-0.350; *p*=0.727
Instruction level (years)	7.19 ± 3.54	10.25 ± 4.17	T_{73}=3.348; *p*=0.001	9.56 ± 4.72	10.96 ± 4.79	T_{73}=1.199; *p*=0.234
Gender	24 f and 8 m	31 f and 12 m	χ^2= 0.079; *p*=0.778	19 f and 6 m	27 f and 23 m	χ^2= 3.401; *p*=0.065
PASE (score)	112.84 ± 31.05	111.65 ± 28.23	T_{73}=-0.173; *p*=0.863	117.4 ± 34.08	110.06 ± 29.19	T_{73}=-0.970; *p*=0.335

Values are means ± SDs

SMC = subjective memory complaints; MCI = mild cognitive impairment;

* the comparisons in baseline concerning age, instruction levels and PASE scores between EG and CG for the 3 categories, were tested using T-test; the homogeneity concerning gender distribution was instead tested using chi-square (χ^2).

Table 2S. AIC (Akaike Information Criterion) and BIC (Bayesian Information Criterion) values used to determine the optimal covariate structure for LMM analysis.

Category A - Healthy participants

	AR1 AIC	AR1 BIC	ARH1 AIC	ARH1 BIC	CS AIC	CS BIC	CSH AIC	CSH BIC	UN AIC	UN BIC	USED IN ANALYSIS
**MMSE**	3788.581	3818.428	3674.089	3723.834	3756.532	3786.379	3617.447	3667.192	3701.375	3775.992	**ARH1**
**FAB**	3283.39	3313.237	3230.372	3280.116	3155.537	3185.384	3118.885	3168.63	3111.321	3185.939	**ARH1**
**RAVLT-i**	7853.204	7883.05	7859.312	7909.057	7551.684	7581.531	7552.98	7602.724	7545.477	7620.094	**AR1**
**RAVLT-d**	5167.879	5197.726	5173.463	5223.208	4881.506	4911.353	4881.175	4930.919	4875.113	4949.73	**AR1**
**AMT**	6893.542	6923.389	6899.646	6949.391	6448.97	6478.816	6445.875	6495.619	6423.009	6497.626	**AR1**
**CPM**	6272.987	6302.834	6279.234	6328.978	5812.666	5842.513	5814.034	5863.779	5774.766	5849.384	**AR1**
**SWCT-e**	3241.543	3271.39	3081.685	3131.429	3153.39	3183.236	2956.725	3006.47	2996.608	3071.225	**ARH1**
**SWCT-t**	8293.838	8323.685	8283.786	8333.531	8043.186	8073.033	8010.577	8060.322	7944.16	8018.777	**AR1**
**PMT-i**	3340.4	3370.247	3327.128	3376.872	3176.635	3206.482	3142.661	3192.405	3148.124	3222.742	**AR1**
**PMT-d**	3710.402	3740.248	3699.892	3749.637	3594.647	3624.493	3576.401	3626.146	3590.759	3665.376	**AR1**
**TMT**	10923.6	10953.45	10911.65	10961.4	10684.84	10714.69	10688.82	10738.56	10300.61	10375.23	**AR1**
**CGD-wo**	4216.052	4245.899	4204.759	4254.504	3986.623	4016.47	3954.832	4004.577	3955.37	4029.987	**AR1**
**CGD-w**	5012.411	5042.258	4898.402	4948.147	4805.905	4835.752	4644.919	4694.664	4727.299	4801.916	**ARH1**

Category B - Participants with SMC

	AR1 AIC	AR1 BIC	ARH1 AIC	ARH1 BIC	CS AIC	CS BIC	CSH AIC	CSH BIC	UN AIC	UN BIC	USED IN ANALYSIS
**MMSE**	3057.508	3086.435	3064.886	3113.098	2927.276	2956.203	2933.312	2981.523	2907.229	2979.546	**AR1**
**FAB**	3094.266	3123.192	3088.276	3136.487	2958.49	2987.417	2961.101	3009.312	2882.367	2954.684	**AR1**
**RAVLT-i**	6829.054	6857.981	6834.977	6883.188	6576.432	6605.358	6582.486	6630.697	6556.983	6629.3	**AR1**
**RAVLT-d**	4617.226	4646.152	4621.298	4669.509	4352.367	4381.293	4348.49	4396.701	4333.119	4405.435	**AR1**
**AMT**	6032.499	6061.425	6038.255	6086.466	5711.748	5740.675	5703.076	5751.287	5685.704	5758.021	**AR1**
**CPM**	5479.254	5508.181	5481.942	5530.153	5164.253	5193.179	5150.592	5198.803	5127.317	5199.633	**AR1**
**SWCT-e**	2905.432	2934.358	2898.712	2946.923	2826.537	2855.464	2814.226	2862.437	2826.186	2898.503	**AR1**

**SWCT-t**	6989.747	7018.674	6991.36	7039.571	*6822.866*	*6851.792*	*6805.871*	*6854.082*	*6784.051*	*6856.367*	**AR1**
**PMT-i**	3119.029	3147.955	3090.741	3138.952	*2935.479*	*2964.406*	*2883.445*	*2931.656*	*2927.732*	*3000.049*	**ARH1**
**PMT-d**	3278.501	3307.427	3264.817	3313.028	*3174.984*	*3203.91*	*3155.895*	*3204.107*	*3172.728*	*3245.044*	**AR1**
**TMT**	9140.219	9169.146	9115.14	9163.351	*8969.765*	*8998.691*	*8926.687*	*8974.898*	*8642.671*	*8714.988*	**ARH1**
**CGD-wo**	4004.144	4033.07	3991.574	4039.785	*3632.699*	*3661.625*	*3604.499*	*3652.71*	*3631.925*	*3704.242*	**AR1**
**CGD-w**	4539.083	4568.009	4530.209	4578.421	*4261.368*	*4290.294*	*4242.438*	*4290.649*	*4216.759*	*4289.076*	**AR1**

Category C - Participants with MCI

	**AR1 AIC**	**AR1 BIC**	**ARH1 AIC**	**ARH1 BIC**	**CS AIC**	**CS BIC**	**CSH AIC**	**CSH BIC**	**UN AIC**	**UN BIC**	**USED IN ANALYSIS**
**MMSE**	1488.089	1511.603	1485.766	1524.955	*1401.927*	*1425.44*	*1388.303*	*1427.492*	*1369.143*	*1427.926*	**AR1**
**FAB**	1461.674	1485.188	1454.025	1493.214	*1361.901*	*1385.415*	*1340.217*	*1379.406*	*1356.317*	*1415.101*	**AR1**
**RAVLT-i**	2660.934	2684.447	2648.348	2687.537	*2567.892*	*2591.405*	*2561.762*	*2600.951*	*2553.695*	*2612.478*	**AR1**
**RAVLT-d**	1415.64	1439.153	1416.036	1455.225	*1399.628*	*1423.141*	*1397.888*	*1437.077*	*1405.563*	*1464.346*	**AR1**
**AMT**	2566.88	2590.394	2563.028	2602.217	*2333.046*	*2356.559*	*2308.66*	*2347.848*	*2303.58*	*2362.363*	**AR1**
**CPM**	2334.575	2358.088	2324.521	2363.71	*2158.296*	*2181.81*	*2120.321*	*2159.51*	*2157.173*	*2215.957*	**AR1**
**SWCT-e**	1361.287	1384.8	1364.716	1403.905	*1227.716*	*1251.229*	*1219.922*	*1259.11*	*1219.709*	*1278.492*	**AR1**
**SWCT-t**	3137.432	3160.945	3126.444	3165.633	*2934.924*	*2958.438*	*2916.063*	*2955.251*	*2911.012*	*2969.796*	**AR1**
**PMT-i**	1386.377	1409.89	1392.633	1431.822	*1323.702*	*1347.216*	*1328.931*	*1368.12*	*1315.817*	*1374.6*	**AR1**
**PMT-d**	1483.333	1506.847	1477.253	1516.442	*1428.546*	*1452.06*	*1411.253*	*1450.442*	*1426.509*	*1485.292*	**AR1**
**TMT**	4077.338	4100.851	3944.933	3984.122	*4050.508*	*4074.021*	*3854.866*	*3894.055*	*3699.217*	*3758.001*	**ARH1**
**CGD-wo**	1660.32	1683.833	1656.519	1695.708	*1498.038*	*1521.551*	*1483.563*	*1522.752*	*1508.383*	*1567.167*	**AR1**
**CGD-w**	2130.011	2153.524	*2094.698*	*2133.887*	*1973.975*	*1997.489*	*1894.05*	*1933.239*	*1926.675*	*1985.458*	**AR1**

Note: Values in italics should be considered invalid because the corresponding LMM estimations yielded non-positive definite Hessian matrices or failed to converge. The Model selection was guided primarily by the Bayesian Information Criterion (BIC), which imposes a conservative penalty on model complexity proportional to the sample size, thereby favoring parsimonious structures that enhance the generalizability and stability of fixed-effect estimates. Compound symmetry (CS), heterogeneous compound symmetry (CSH), and unstructured (UN) covariance structures were also tested; however, they were excluded because, as previously stated, they resulted in a non-positive definite final Hessian matrix. ARH1 also resulted in an invalid analysis for CGD-W in MCI category. Consequently, only covariance structures that achieved full convergence and a positive Hessian matrix were considered.

Table 3S. Full LMM results for the three categories, including F-values, p-values, and partial eta-squared for all tests, including non-significant ones.

**Category A - Healthy participants**			
	**Group**	**Time**	**Time*groups**
**MMSE**	$F_{1,1063}=6.992$; $p=0.008$; $\eta^2_p=0.007$	$F_{4,1063}=19.051$; $p<0.001$; $\eta^2_p=0.067$	$F_{4,1063}=6.915$; $p<0.001$; $\eta^2_p=0.025$
**FAB**	$F_{1,1063}=7.871$; $p=0.005$; $\eta^2_p=0.007$	$F_{4,1063}=3.924$; $p=0.004$; $\eta^2_p=0.015$	$F_{4,1063}=3.720$; $p=0.005$; $\eta^2_p=0.014$
**RAVLT-i**	$F_{1,1063}=5.960$; $p=0.015$; $\eta^2_p=0.006$	$F_{4,1063}=6.536$; $p<0.001$; $\eta^2_p=0.024$	$F_{4,1063}=4.243$; $p=0.002$; $\eta^2_p=0.016$
**RAVLT-d**	$F_{1,1063}=22.290$; $p<0.001$; $\eta^2_p=0.021$	$F_{4,1063}=6.581$; $p<0.001$; $\eta^2_p=0.024$	$F_{4,1063}=6.813$; $p<0.001$; $\eta^2_p=0.025$
**AMT**	$F_{1,1063}=8.150$; $p=0.004$; $\eta^2_p=0.008$	$F_{4,1063}=0.989$; $p=0.413$; $\eta^2_p=0.004$ (NS)	$F_{4,1063}=1.454$; $p=0.214$; $\eta^2_p=0.005$ (NS)
**CPM**	$F_{1,1063}=8.975$; $p=0.003$; $\eta^2_p=0.008$	$F_{4,1063}=9.574$; $p<0.001$; $\eta^2_p=0.035$	$F_{4,1063}=4.333$; $p=0.002$; $\eta^2_p=0.016$
**SWCT-e**	$F_{1,1063}=4.707$; $p=0.030$; $\eta^2_p=0.004$ (NSA)	$F_{4,1063}=3.063$; $p=0.016$; $\eta^2_p=0.011$	$F_{4,1063}=0.805$; $p=0.522$; $\eta^2_p=0.003$ (NS)
**SWCT-t**	$F_{1,1063}=0.131$; $p=0.718$; $\eta^2_p<0.001$ (NS)	$F_{4,1063}=4.564$; $p=0.001$; $\eta^2_p=0.017$	$F_{4,1061}=0.609$; $p=0.656$; $\eta^2_p=0.002$ (NS)
**PMT-i**	$F_{1,1063}=35.212$; $p<0.001$; $\eta^2_p=0.032$	$F_{4,1063}=5.645$; $p<0.001$; $\eta^2_p=0.021$	$F_{4,1063}=12.972$; $p<0.001$; $\eta^2_p=0.047$
**PMT-d**	$F_{1,1063}=29.590$; $p<0.001$; $\eta^2_p=0.027$	$F_{4,1063}=6.263$; $p<0.001$; $\eta^2_p=0.023$	$F_{4,1063}=8.971$; $p<0.001$; $\eta^2_p=0.033$
**TMT**	$F_{1,1047}=1.511$; $p=0.219$; $\eta^2_p=0.001$ (NS)	$F_{4,1047}=4.436$; $p=0.001$; $\eta^2_p=0.017$	$F_{4,1047}=3.468$; $p=0.008$; $\eta^2_p=0.013$
**CGD-wo**	$F_{1,1063}=0.001$; $p=0.981$; $\eta^2_p<0.001$ (NS)	$F_{4,1063}=2.508$; $p=0.040$; $\eta^2_p=0.009$	$F_{4,1063}=1.347$; $p=0.251$; $\eta^2_p=0.005$ (NS)
**CGD-w**	$F_{1,1063}=0.358$; $p=0.839$; $\eta^2_p<0.001$ (NS)	$F_{4,1063}=6.699$; $p<0.001$; $\eta^2_p=0.025$	$F_{4,1063}=0.358$; $p=0.839$; $\eta^2_p=0.001$ (NS)

**Category B - Participants with SMC**			
	**Group**	**Time**	**Time*groups**
**MMSE**	$F_{1,911}=3.785$; $p=0.052$; $\eta^2_p=0.004$ (NS)	$F_{4,911}=8.381$; $p<0.001$; $\eta^2_p=0.035$	$F_{4,911}=7.281$; $p<0.001$; $\eta^2_p=0.031$
**FAB**	$F_{1,911}=1.286$; $p=0.257$; $\eta^2_p=0.001$ (NS)	$F_{4,911}=2.804$; $p=0.025$; $\eta^2_p=0.012$	$F_{4,911}=1.208$; $p=0.306$; $\eta^2_p=0.005$ (NS)
**RAVLT-i**	$F_{1,911}=9.086$; $p=0.003$; $\eta^2_p=0.010$	$F_{4,911}=6.173$; $p<0.001$; $\eta^2_p=0.026$	$F_{4,911}=6.548$; $p<0.001$; $\eta^2_p=0.028$
**RAVLT-d**	$F_{1,911}=8.374$; $p=0.004$; $\eta^2_p=0.009$	$F_{4,911}=4.322$; $p=0.002$; $\eta^2_p=0.019$	$F_{4,911}=4.273$; $p=0.002$; $\eta^2_p=0.018$
**AMT**	$F_{1,911}=8.146$; $p=0.004$; $\eta^2_p=0.009$	$F_{4,911}=7.924$; $p<0.001$; $\eta^2_p=0.034$	$F_{4,911}=6.964$; $p<0.001$; $\eta^2_p=0.030$
**CPM**	$F_{1,915}=7.975$; $p=0.005$; $\eta^2_p=0.009$	$F_{4,911}=6.549$; $p<0.001$; $\eta^2_p=0.028$	$F_{4,911}=6.137$; $p<0.001$; $\eta^2_p=0.026$
**SWCT-e**	$F_{1,911}=0.919$; $p=0.338$; $\eta^2_p=0.001$ (NS)	$F_{4,911}=0.481$; $p=0.750$; $\eta^2_p=0.002$ (NS)	$F_{4,911}=0.600$; $p=0.663$; $\eta^2_p=0.003$ (NS)

**SWCT-t**	$F_{1,911}=3.765$; $p=0.053$; $\eta^2_p=0.004$ (NS)	$F_{4,911}=0.689$; $p=0.600$; $\eta^2_p=0.003$ (NS)	$F_{4,911}=1.702$; $p=0.147$; $\eta^2_p=0.007$ (NS)
**PMT-i**	$F_{1,911}=11.362$; $p=0.001$; $\eta^2_p=0.012$	$F_{4,911}=9.572$; $p<0.001$; $\eta^2_p=0.040$	$F_{4,911}=9.465$; $p<0.001$; $\eta^2_p=0.040$
**PMT-d**	$F_{1,911}=15.784$; $p<0.001$; $\eta^2_p=0.017$	$F_{4,911}=8.267$; $p<0.001$; $\eta^2_p=0.035$	$F_{4,911}=10.798$; $p<0.001$; $\eta^2_p=0.045$
**TMT**	$F_{1,898}=1.292$; $p=0.256$; $\eta^2_p=0.001$ (NS)	$F_{4,898}=5.254$; $p<0.001$; $\eta^2_p=0.023$	$F_{4,898}=0.995$; $p=0.410$; $\eta^2_p=0.004$ (NS)
**CGD-wo**	$F_{1,912}=0.166$; $p=0.683$; $\eta^2_p<0.001$ (NS)	$F_{4,911}=8.707$; $p<0.001$; $\eta^2_p=0.037$	$F_{4,911}=0.752$; $p=0.557$; $\eta^2_p=0.003$ (NS)
**CGD-w**	$F_{1,911}=0.059$; $p=0.809$; $\eta^2_p<0.001$ (NS)	$F_{4,911}=4.568$; $p=0.001$; $\eta^2_p=0.020$	$F_{4,911}=0.546$; $p=0.702$; $\eta^2_p=0.002$ (NS)

**Category C - Participants with MCI**			
	**Group**	**Time**	**Time*groups**
**MMSE**	$F_{1,366}=1.890$; $p=0.170$; $\eta^2_p=0.005$ (NS)	$F_{4,366}=2.602$; $p=0.036$; $\eta^2_p=0.028$	$F_{4,366}=1.169$; $p=0.324$; $\eta^2_p=0.013$ (NS)
**FAB**	$F_{1,366}=0.525$; $p=0.469$; $\eta^2_p=0.001$ (NS)	$F_{4,366}=4.085$; $p=0.003$; $\eta^2_p=0.043$	$F_{4,366}=1.035$; $p=0.389$; $\eta^2_p=0.011$ (NS)
**RAVLT-i**	$F_{1,366}=0.698$; $p=0.404$; $\eta^2_p=0.002$ (NS)	$F_{4,366}=22.576$; $p<0.001$; $\eta^2_p=0.198$	$F_{4,366}=0.733$; $p=0.570$; $\eta^2_p=0.008$ (NS)
**RAVLT-d**	$F_{1,366}=0.705$; $p=0.402$; $\eta^2_p=0.002$ (NS)	$F_{4,366}=21.821$; $p<0.001$; $\eta^2_p=0.193$	$F_{4,366}=0.792$; $p=0.531$; $\eta^2_p=0.009$ (NS)
**AMT**	$F_{1,366}=0.447$; $p=0.504$; $\eta^2_p=0.001$ (NS)	$F_{4,366}=3.385$; $p=0.010$; $\eta^2_p=0.036$	$F_{4,366}=5.461$; $p<0.001$; $\eta^2_p=0.056$
**CPM**	$F_{1,366}=0.327$; $p=0.568$; $\eta^2_p=0.001$ (NS)	$F_{4,366}=5.604$; $p<0.001$; $\eta^2_p=0.058$	$F_{4,366}=1.715$; $p=0.146$; $\eta^2_p=0.018$ (NS)
**SWCT-e**	$F_{1,366}=4.556$; $p=0.033$; $\eta^2_p=0.012$ (NSA)	$F_{4,366}=1.280$; $p=0.277$; $\eta^2_p=0.014$ (NS)	$F_{4,366}=1.040$; $p=0.386$; $\eta^2_p=0.011$ (NS)
**SWCT-t**	$F_{1,366}=0.001$; $p=0.982$; $\eta^2_p<0.001$ (NS)	$F_{4,366}=1.694$; $p=0.151$; $\eta^2_p=0.018$ (NS)	$F_{4,366}=5.226$; $p<0.001$; $\eta^2_p=0.054$
**PMT-i**	$F_{1,366}=0.125$; $p=0.724$; $\eta^2_p<0.001$ (NS)	$F_{4,366}=7.300$; $p<0.001$; $\eta^2_p=0.074$	$F_{4,366}=4.187$; $p=0.003$; $\eta^2_p=0.044$
**PMT-d**	$F_{1,366}=0.059$; $p=0.808$; $\eta^2_p<0.001$ (NS)	$F_{4,366}=15.770$; $p<0.001$; $\eta^2_p=0.147$	$F_{4,366}=7.098$; $p<0.001$; $\eta^2_p=0.072$
**TMT**	$F_{1,360}=5.190$; $p=0.023$; $\eta^2_p=0.014$ (NSA)	$F_{4,360}=4.432$; $p=0.002$; $\eta^2_p=0.047$	$F_{4,360}=1.547$; $p=0.188$; $\eta^2_p=0.017$ (NS)
**CGD-wo**	$F_{1,366}=0.100$; $p=0.752$; $\eta^2_p<0.001$	$F_{4,366}=4.082$; $p=0.003$; $\eta^2_p=0.043$	$F_{4,366}=5.544$; $p<0.001$; $\eta^2_p=0.057$
**CGD-w**	$F_{1,366}=0.221$; $p=0.639$; $\eta^2_p=0.001$	$F_{4,366}=8.925$; $p<0.001$; $\eta^2_p=0.089$	$F_{4,366}=2.125$; $p=0.077$; $\eta^2_p=0.023$ (NS)

(NS), Not significant; (NSA), Not significant after False Discovery Rate adjustment for multiple comparisons (Benjamini-Hochberg method); MMSE, Mini Mental State Examination; FAB, Frontal Assessment Battery; RAVLT, Rey's Auditory Verbal Learning Test; PMT, Prose Memory Test; AMT, Attentive Matrices Test; CPM, Raven's Colored Progressive Matrices; SWCT, Stroop Word-Color Interference test; TMT, Trail Making Test; CGD, Copying Geometric Drawings. EG, experimental group; CG, control group; -i, immediate recall; -d, delayed recall; -e, errors; -t, time; -w, with landmarks; -wo, without landmarks

Paragraph 1S. Mechanism of Missingness in Longitudinal Data: Evidence for a Missing at Random (MAR) Process

To investigate the mechanism underlying the missing data in the dataset across the study period, several diagnostic analyses were performed. Overall, missingness increased progressively over time, rising from 0% at baseline to 25.9% at the 12-month assessment, 36.6% at 24 months, 31.8% at 36 months, and reaching 54.0% at the 48-month assessment. Little's Missing Completely at Random (MCAR) test was highly significant ($\chi^2 = 134.1$, df = 38, $p < 0.001$), strongly rejecting the hypothesis that data were missing completely at random. To evaluate whether the missingness pattern adhered to a Missing at Random (MAR) mechanism, a Cox proportional hazards regression model was conducted to predict time to dropout based on baseline characteristics (Group, Age, Educational Level, and baseline MMSE). The analysis revealed that dropout was significantly predicted by fully observed baseline variables. Specifically, group assignment was a highly significant predictor ($p < 0.001$); patients in the Experimental Group (EG) exhibited a significantly lower risk of dropping out compared to those in the Control Group (CG) (HR = 0.68). Educational level was also significantly associated with dropout time (p=0.005, HR=1.03). Finally, a pattern-mixture analysis evaluating baseline MMSE scores across different dropout cohorts showed a statistically significant difference (ANOVA, p=0.0014), indicating a slight clinical variation between completers and early dropouts. Taken together, because the missingness and dropout patterns were systematically associated with observed baseline characteristics (group assignment and educational level), these findings strongly support a Missing at Random (MAR) mechanism. Consequently, subsequent longitudinal analyses were conducted using statistical frameworks robust to MAR data (such as linear mixed-effects models or multiple imputation) to prevent biased estimations. Also, as shown in Table 1S – part 2, a direct comparison of baseline characteristics between completers (analysed) and dropouts (not analysed) was performed. The results indicate that the two groups were homogeneous across almost all variables, with the sole significant exception of age within the EG.
